# Establishment and Validation of Novel Prognostic Subtypes in Hepatocellular Carcinoma Based on Bile Acid Metabolism Gene Signatures Using Bulk and Single-Cell RNA-Seq Data

**DOI:** 10.3390/ijms25020919

**Published:** 2024-01-11

**Authors:** Yimo Qu, Xiaocheng Gong, Ziyuan Zhao, Zimei Zhang, Qian Zhang, Yuting Huang, Qingsong Xie, Yunfei Liu, Jinfen Wei, Hongli Du

**Affiliations:** School of Biology and Biological Engineering, South China University of Technology, Guangzhou Higher Education Mega Centre, Panyu District, Guangzhou 510006, China; quym796@163.com (Y.Q.); 202010108425@mail.scut.edu.cn (X.G.); 202221049038@mail.scut.edu.cn (Z.Z.); 202110189419@mail.scut.edu.cn (Z.Z.); 202110189331@mail.scut.edu.cn (Q.Z.); ythuang907@163.com (Y.H.); 202021049131@mail.scut.edu.cn (Q.X.); 202120149279@mail.scut.edu.cn (Y.L.)

**Keywords:** hepatocellular carcinoma, bile acids metabolism, tumor subtypes, prognosis, medication guidance, cell cycle

## Abstract

Hepatocellular carcinoma (HCC) is a highly detrimental cancer type and has limited therapeutic options, posing significant threats to human health. The development of HCC has been associated with a disorder in bile acid (BA) metabolism. In this study, we employed an integrative approach, combining various datasets and omics analyses, to comprehensively characterize the tumor microenvironment in HCC based on genes related to BA metabolism. Our analysis resulted in the classification of HCC samples into four subtypes (C1, C2a, C2b, and C3). Notably, subtype C2a, characterized by the highest bile acid metabolism score (BAMS), exhibited the highest survival probability. This subtype also demonstrated increased immune cell infiltration, lower cell cycle scores, reduced AFP levels, and a lower risk of metastasis compared to subtypes C1 and C3. Subtype C1 displayed poorer survival probability and elevated cell cycle scores. Importantly, the identified subtypes based on BAMS showed potential relevance to the gene expression of drug targets in currently approved drugs and those under clinical research. Genes encoding *VEGFR* (FLT4 and KDR) and *MET* were elevated in C2, while genes such as *TGFBR1*, *TGFB1*, *ADORA3*, *SRC*, *BRAF*, *RET*, *FLT3*, *KIT*, *PDGFRA*, and *PDGFRB* were elevated in C1. Additionally, *FGFR2* and *FGFR3*, along with immune target genes including *PDCD1* and *CTLA4*, were higher in C3. This suggests that subtypes C1, C2, and C3 might represent distinct potential candidates for *TGFB1* inhibitors, *VEGFR* inhibitors, and immune checkpoint blockade treatments, respectively. Significantly, both bulk and single-cell transcriptome analyses unveiled a negative correlation between BA metabolism and cell cycle-related pathways. In vitro experiments further confirmed that the treatment of HCC cell lines with BA receptor agonist ursodeoxycholic acid led to the downregulation of the expression of cell cycle-related genes. Our findings suggest a plausible involvement of BA metabolism in liver carcinogenesis, potentially mediated through the regulation of tumor cell cycles and the immune microenvironment. This preliminary understanding lays the groundwork for future investigations to validate and elucidate the specific mechanisms underlying this potential association. Furthermore, this study provides a novel foundation for future precise molecular typing and the design of systemic clinical trials for HCC therapy.

## 1. Introduction

Hepatocellular carcinoma (HCC) stands as a formidable cancer, ranking as the second leading cause of cancer-related deaths globally [1]. Over 80% of HCC cases emerge within the backdrop of chronic liver diseases stemming from factors such as viral hepatitis, alcohol abuse, obesity, diabetes, and metabolic syndrome [2,3]. This diverse etiology and varied natural history contribute to the pronounced heterogeneity of HCC, posing significant challenges to clinical management [4]. The escalating incidence of nonalcoholic fatty liver disease, spanning from steatosis to advanced cirrhosis in nonalcoholic steatohepatitis [3], has elevated metabolic dysfunction as a prominent risk factor for HCC development [2,5]. Importantly, these metabolic aberrations are also associated with heightened risks of liver and colorectal cancers [6]. Consequently, understanding the mechanisms of metabolic reprogramming in cancer development is crucial for exploring novel targeting strategies and overcoming current limitations in systemic therapy for HCC.

As our comprehension of tumor biology and metabolism advances, metabolic reprogramming emerges as a hallmark of malignancy. Notably, various metabolic disturbances, including glucose, fatty acid, and amino acid metabolism, have been reported in HCC, leading to the development of medications targeting dysregulated metabolism [7]. Particularly, bile acid (BA) metabolism’s role in the development and progression of HCC has been confirmed in previous studies. Generally, the biosynthesis, metabolism, and excretion of BAs occur in the liver, and the homeostasis of BAs in the body is tightly regulated with regard to cellular and tissue concentrations [8]. Studies show that BAs are natural ligands for a variety of nuclear and membrane receptors, such as the farnesoid X receptor (FXR), pregnane X receptor (PXR), vitamin D receptor (VDR), and the G protein-coupled bile acid receptor 5 (TGR5), engaged in the regulation of lipid, glucose, energy, and drug metabolism as essential signaling molecules [9], and they have emerged as an attractive etiologic driver and therapeutic target for intrahepatic cholestasis and metabolic liver disease [10,11]. For instance, ursodeoxycholic acid (UDCA) is approved as a first-line treatment for primary biliary cholangitis and has demonstrated efficacy in dissolving cholesterol gallstones and improving liver function in cholestatic diseases [12].

Significantly, the disruption of BA homeostasis has been identified as a clinical hallmark of HCC through metabolome analysis in biological fluids [13,14]. A multi-center study based on metabolomics identified long-term elevated serum BAs but decreased levels of glycocholate, glycochenodeoxycholate, and glycoursodeoxycholate in HCC patients compared with the normal controls [15]. Pathway analysis, utilizing differential metabolites, demonstrated that metabolic reprogramming in HCC was primarily associated with primary BAs biosynthesis, glycerophospholipid metabolism, and tryptophan metabolism [16]. However, the integration of BAs metabolism with other biological processes and its impact on HCC progression remain unclear. Furthermore, the potential utility of BA metabolism-related genes in identifying prognostic molecular subtypes of HCC patients has yet to be explored.

To address these gaps, we quantified BA metabolism using single-sample gene set enrichment analysis (ssGSEA) and established a BA metabolism-related genes enrichment score (BAMS) to assess its perturbation in HCC. Utilizing the Cancer Genome Atlas-Liver Hepatocellular Carcinoma (TCGA-LIHC) cohort as the training dataset (including 368 tumor samples), we employed non-negative matrix factorization (NMF) methods to identify novel BA metabolism-based subtypes of HCC. External validation of the classification was performed using GEO datasets, which included 733 and 231 HCC tumor samples. Additionally, single-cell transcriptome data from three GEO datasets, encompassing 49 HCC samples, were incorporated. We systematically explored the integrated association between BA metabolism and its prognostic value, clinical characteristics, transcriptome features, and other pathways. Comparative analyses were conducted with previously recognized HCC subtypes and current drug targets, potentially providing deeper insights into molecular classification and guiding medication strategies for liver cancer. Notably, the relationship between BA metabolism and the cell cycle was experimentally verified in vitro, introducing a speculative dimension that may inform future therapeutic strategies in HCC.

## 2. Results

### 2.1. Altered BA Metabolism Associated with Cancer Biological Pathways, Clinical Prognosis, and Advanced Progression

**Figure 1:**
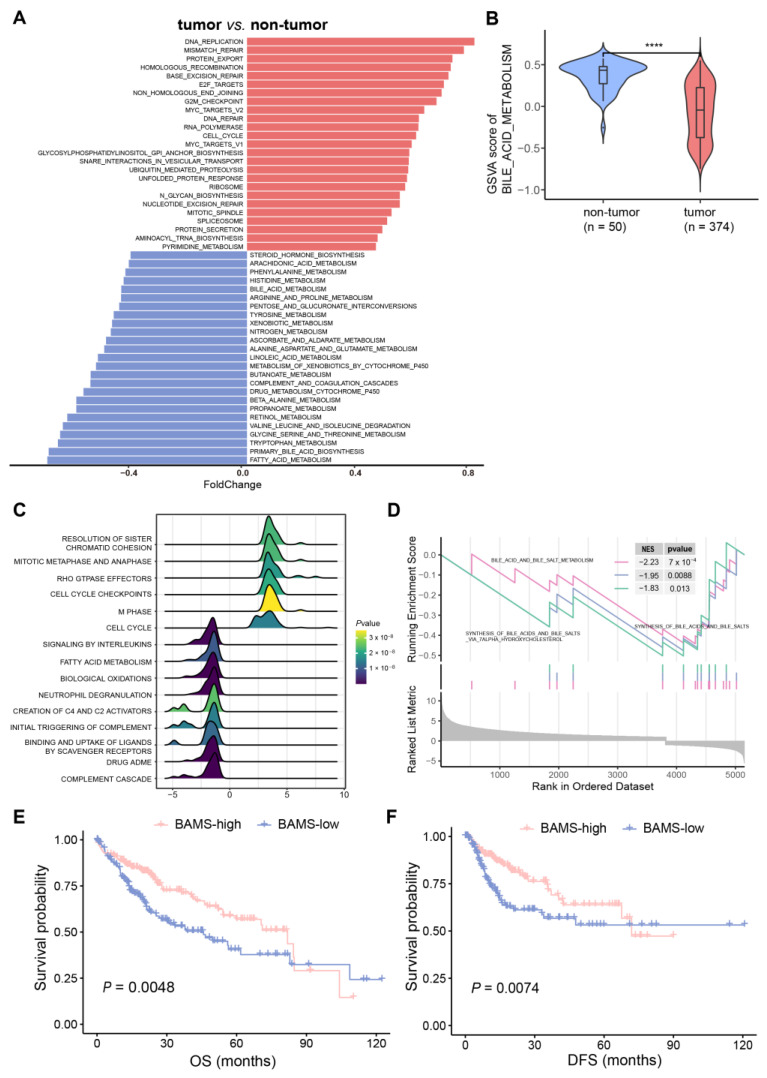
Characteristics of bile acid metabolism in hepatocellular carcinoma (HCC). (**A**) Alteration pathways in the samples from the tumor (n = 374) versus non-tumor (n = 50) samples of TCGA-LIHC cohort. Upregulated and downregulated pathways are indicated in red (**right**) and blue (**left**) bars. Foldchange was calculated using the R package “limma (v. 3.58.1)”. (**B**) Gene set variation analysis (GSVA) showed that bile acid metabolism was downgraded in HCC compared to the non-tumor samples. In the violin plots, the middle bar represents the median, and the box represents the interquartile range (the 25th and 75th percentiles); the vertical line denoted 1.5 times the interquartile range. *p*-values are calculated via the Wilcoxon rank-sum test (**** *p* < 0.0001). The results of GSEA analysis illustrated in (**C**) the top 15 pathways with the largest |NES| and (**D**) BA metabolism-related gene set enrichment. Kaplan–Meier curves of overall survival (OS) (**E**) and disease-free survival (DFS) (**F**) for BAMS-high and BAMS-low of TCGA-LIHC cohort.

GSVA analysis was performed on 374 HCC and 50 non-tumor samples based on the 236 gene sets (Supplementary Table S1). To further address whether BA metabolism is altered in HCC at the transcriptome level, we conducted a differential pathway analysis and found that metabolic disturbances were mainly related to BA metabolism, fatty acid metabolism, and glycine/serine/threonine metabolism. In addition, abnormal activity of cell cycle-related pathways was found in HCC, such as “DNA_REPLICATION”, “MISMATCH_REPAIR”, “BASE_EXCISION_REPAIR”, and “E2F_TARGETS” (Figure 1A). Notably, BA metabolism showed a significant reduction in HCC compared with non-tumor samples (Figure 1B), which were further validated in three external training cohorts (GSE14520, GSE25097, and GSE36376) (Supplementary Figure S1A,B). GSEA also indicated that cell cycle-related pathways were enriched in HCC, while BA metabolism-related pathways showed the opposite trend (Figure 1B). We then performed differential gene expression analysis between tumor and non-tumor samples to identify BA metabolism-related genes. BA metabolism-related genes were obtained from six gene sets and filtered using differentially expressed genes in HCC (Supplementary Table S2). Furthermore, 95 BA metabolism-related genes were screened and defined as the BAs metabolism gene set for calculating the bile acid metabolism-related enrichment score (BAMS) via ssGSEA (Supplementary Table S2). BAMS-high and -low were grouped using the median of BAMS to further investigate whether BAMS could predict the prognosis of HCC. Kaplan–Meier analysis showed that BAMS-high was associated with improved overall survival (OS) (*p* = 0.0048, Figure 1D) and disease-free survival (DFS) (*p* = 0.0074, Figure 1D) compared with BAMS-low, which was also found in another dataset (*p* = 0.0014, Supplementary Figure S1C). Consistently, BAMS was significantly different among clinical stages of HCC, with lower BAMS indicating more advanced HCC (Supplementary Figure S2A). These findings suggest a hypothetical link between altered BA metabolism, cell cycle dysregulation, and the clinical outcomes of HCC, warranting further exploration and validation in future studies.

### 2.2. Three Distinct Subtypes in HCC Identified Based on BA Metabolism-Related Genes Using NMF Methods

There were 95 BA metabolism-related genes subjected to NMF in R for unsupervised consensus clustering. The rank survey profiles of the cophenetic score and dispersion suggested that there were three distinct molecular subtypes of HCC with different BA metabolism-related gene expression patterns (Supplementary Figure S2B), with C1, n = 51; C2, n = 190; and C3, n = 127. Two-dimensional t-SNE distribution based on the mRNA expression of BA metabolism-related genes was performed to validate the assignment of the three subtypes (Supplementary Figure S2C). Subsequently, a differential pathways analysis between the three subtypes was carried out (Supplementary Table S2).

**Figure 2:**
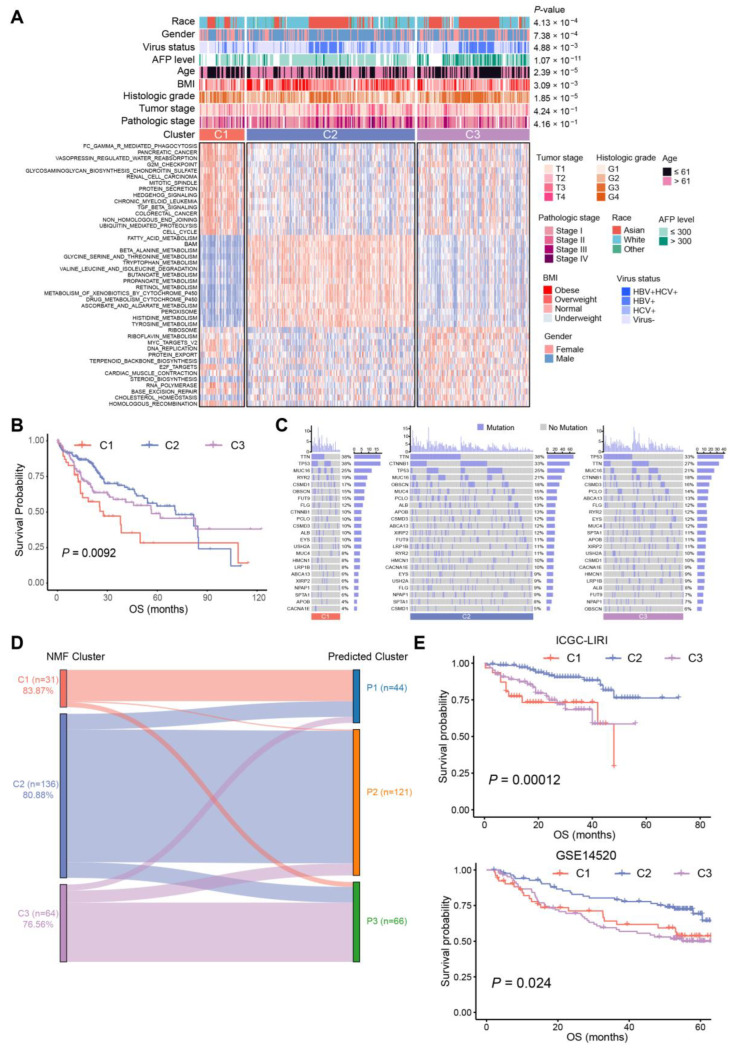
Subtypes of 368 HCC samples and association with clinical characteristics. (**A**) Heat map of alteration pathways in three subtypes identified via non-negative matrix factorization (NMF) consensus clustering (tumor samples, n = 368): C1 (red, n = 51), C2 (blue, n = 190), and C3 (purple, n = 127). Clinical characteristics are shown above with *p*-values calculated using a chi-square test. Underweight: body mass index (BMI) <18.5; normal: 18.5 ≤ BMI ≤ 25; overweight: 25 < BMI ≤ 30; obese: BMI > 30. (**B**) Kaplan–Meier curves of OS for each subtype of TCGA-LIHC cohort. (**C**) Oncoprint of gene mutation in the three subtypes of TCGA-LIHC cohort, purple for mutation and grey for no mutation. (**D**) Comparison of subtypes between NMF clusters and predicted clusters of ICGC-LIRI cohort depicted in the Sankey diagram. The height of each linkage line represents the number of samples. (**E**) Kaplan–Meier curves of OS for subtypes identified by NMF in the ICGC-LIRI cohort (**top**) and predicted subtypes in GSE14520 (**bottom**).

The results showed that C2 was enriched in the metabolism of BAs, fatty acids, retinol, and xenobiotics, while C1 exhibited upper enrichment in pathways associated with tumor progression, and C3 seemed to be in an intermediate position (Figure 2A). Chi-square tests identified significant correlations between clinical characteristics and HCC subtypes. More obese and overweight patients were classified as C2 subtype, and histologic grade G1/G2 was associated with C2 subtype. Patients of the C3 subtype showed a higher AFP level and histologic grade, as well as a higher risk of hepatitis B virus (HBV) infection (Figure 2A, Supplementary Table S3). Consistent with the knowledge that non-proliferative HCCs have a favorable prognosis [17] associated with metabolism of lipids and bile salts, we noticed that C2 exhibited high enrichment in CYP-mediated metabolism and improved prognosis, while C1 and C3 tended to be proliferative HCCs with a poorer prognosis compared to C2 (Figure 2A,B). Meanwhile, C2 contained the highest rate (38%) of *CTNNB1* mutations among the three subtypes, yet C1 had the highest mutation frequency of *TP53* (38%), followed by C3 (33%) (Figure 2C). These results suggested that C2 HCCs were associated with a favorable prognosis, which had the highest level of BA metabolism and the lowest enrichment of cell cycle-related pathways. Subsequently, a correlation analysis of BA metabolism with differential pathways was performed. A significantly negative correlation was seen in BA metabolism and cell cycle-related pathways, such as “MYC_TARGETS_V1”, “CELL_CYCLE”, and “G2M_CHECKPOINT”. The metabolism of fatty acids, and the tryptophan, glycine, serine, and threonine score were positively correlated with the BA metabolism score (Supplementary Figure S2D).

To construct a clinical classifier and validation, the top 30 DEGs ranked by log2FC of each subtype were selected as the subtype marker genes (Supplementary Table S4). Three subtypes were identified in the ICGC-LIRI cohort via NMF in the same way, and the 90-gene classifiers were employed for subclass prediction via nearest template prediction (NTP), with an accuracy of 83.87% in C1, 80.88% in C2, and 76.56% in C3 (Figure 2D). Furthermore, the Kaplan–Meier analysis results of the ICGC-LIRI cohort also confirmed that C2 had improved prognosis compared with C3 and C1 (*p* = 0.00012, Figure 2E), and the result of differential pathways analysis was similar to that of the TCGA cohort (Supplementary Figure S2E). In addition, another dataset (GSE14520) classified by the 90-gene classifier also indicated that the BA metabolism-related gene-based HCC subtypes presented different OS (*p* = 0.024, Figure 2E).

### 2.3. Association between BA Metabolism-Related Subtypes and Previous Subtypes in HCC

**Figure 3:**
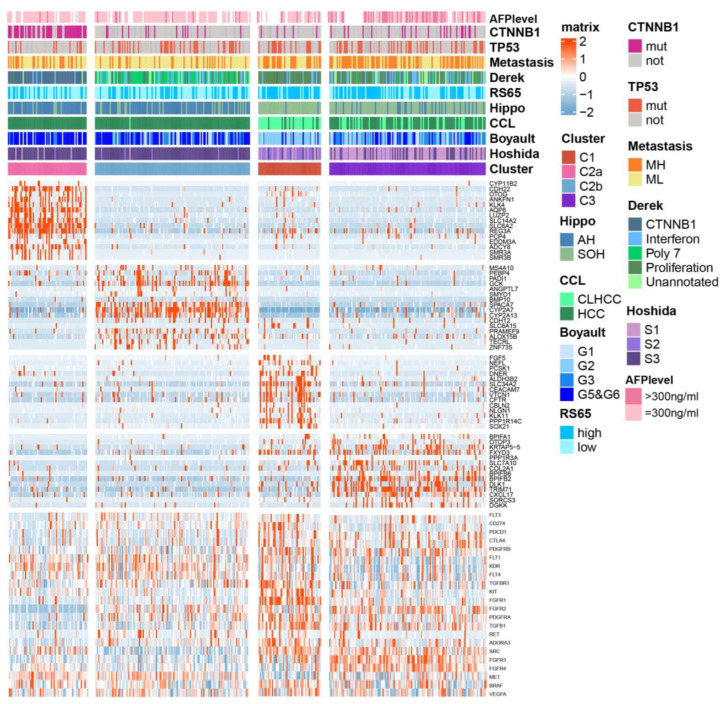
Subtypes of TCGA-LIHC cohort and association with previous HCC subtypes and signatures. Integrated analysis of CTNNB1 and TP53 mutations (**bottom**), DEGs expression data (**middle**), and molecular subtypes of HCC (**top**). (Tumor samples, n = 368): C1 (n = 51), C2a (n =64), C2b (n =126), and C3 (n = 127). Metastasis risk subtype, MH for high risk and ML for low risk; RS65, 65-gene risk score; Hippo, Hippo pathway subtype; AH for active Hippo; SOH for silence of Hippo; CCL, cholangiocarcinoma-like subtype; CLHCC for cholangiocarcinoma-like HCC. *p*-values were calculated using a chi-square test.

Given that the above three subtypes were classified via BA metabolism-related genes, and that C1 and C3 were characterized as proliferative HCC, we then further addressed whether there were subtypes with differential expression patterns of BA metabolism-related genes among C2 belonging to the non-proliferative HCC. C2 was divided into two subtypes, named C2a (n = 64) and C2b (n = 126). A heat map displays the top 15 DEGs for each subtype and the correlation with previous classifications (Figure 3). C2a was significantly correlated with Chiang’s CTNNB1 subclass and had the highest frequency of *CNTTB1* mutation (*p* < 0.001). C2, with the most favorable prognosis, exhibited similarity to the active Hippo (AH) pathway subgroup [18], CCL’s HCC subclass [19], CTNNB1 activating mutations subclass (Boyault’s G5/G6) [20], and the well-differentiated subclass (Hoshida’s S3) [21]. C1 and C3 were more correlated with metastasis high risk (MH) [22], Derek’s proliferation subclass [23], high risk scores of 65-genes (RS65, high) [24], silencing of the Hippo pathway (SOH), and cholangiocarcinoma-like HCC (CLHCC), suggesting that HCC with the C1/C3-like gene expression pattern represents a poor outcome and a more aggressive tumor (Figure 3).

Furthermore, we conducted a comparative analysis of the expression levels of genes corresponding to drug targets that are approved or under clinical trials across the subtypes, including VEGFR, PDGFR, KIT, PD-L1, and PD-1. Remarkably, VEGFR-encoding genes *FLT4* and *KDR*, as well as *MET*, exhibited higher expression in C2 subtypes. On the other hand, *TGFBR1*, *TGFB1*, *ADORA3*, *RET*, *PDGFRA*, and *PDGFRB* were elevated in C1, while *FGFR2* and *FGFR3*, along with immune target genes such as *PDCD1* and *CTLA4*, showed higher expression in C3 (Figure 3, Supplementary Figure S3). These findings suggest that the current subclassification based on BA metabolism might serve as a potential valuable guide for precision medication strategies in HCC treatment.

### 2.4. Biological Characteristics and Immune Infiltration Level between Different Subtypes

**Figure 4:**
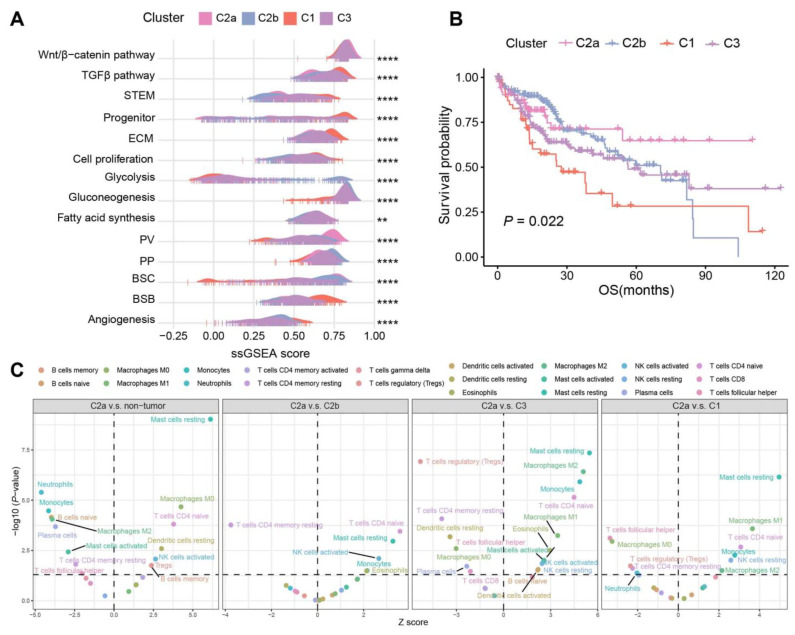
Subtypes and signatures of TCGA-LIHC. (**A**) Association between the four subtypes and HCC signatures depicted in the ridge plot. (**B**) Kaplan–Meier curves of OS for the four subtypes in HCC samples. (**C**) CIBERSORT inferred immune cell populations between C2a, non-tumor samples, and other subtypes. *p* values were calculated via Wilcoxon rank-sum test and *p* < 0.05 was statistically significant (dotted lines on the y axis). Only the significant cell types were labeled. **** *p* < 0.0001, ** *p* < 0.01.

We then assessed the correlation of specific HCC signatures across the four subtypes. As was described previously, C2 was enriched in the metabolism of BAs, fatty acids, and amino acids, which share similar patterns with PP (periportal)-type and PV (perivenous)-type HCCs [25] (Figure 2A). Particularly, C2a presented the highest PV score, which is in line with the study that showed that the PV-type HCCs exhibited a higher mutation frequency of CTNNB1 [25]. By contrast, C1 and C3 showed comparable characteristics as the proliferative HCC, with TGFβ activation, stem cell features, cell cycle progression, higher vascular invasion, and poor prognosis. C2a and C2b had a higher score of bile salt efflux into canaliculi (BSC) than other subtypes, while C1 was most enriched in the bile salt efflux into blood flow (BSB) signature, implying that more bile salts in C2 samples efflux into bile canaliculi and may cause cholestasis [20] (Figure 4A). Also, survival analysis was significantly different among the four subtypes (*p* = 0.022, Figure 4B).

We conducted a detailed investigation into the distinctions in immune cell infiltration among subtypes using the CIBERSORT [26], which estimates the relative proportions of various immune cell types. When comparing C2a with other samples, the proportion of monocytes and M2 macrophages changed from activation in the non-tumor samples to depletion in C1 and C3, while M0 macrophages exhibited the opposite trend. In C2a, we observed increases in mast resting cells, CD4 naive T cells, M1/M2 macrophages, and NK resting cells, and a reduction in regulatory T cells, CD4 memory resting T cells, and T follicular helper cells compared to C1 and C3 (Figure 4C).

### 2.5. BA Metabolism in the Level of Single-Cell Transcriptome Data

**Figure 5:**
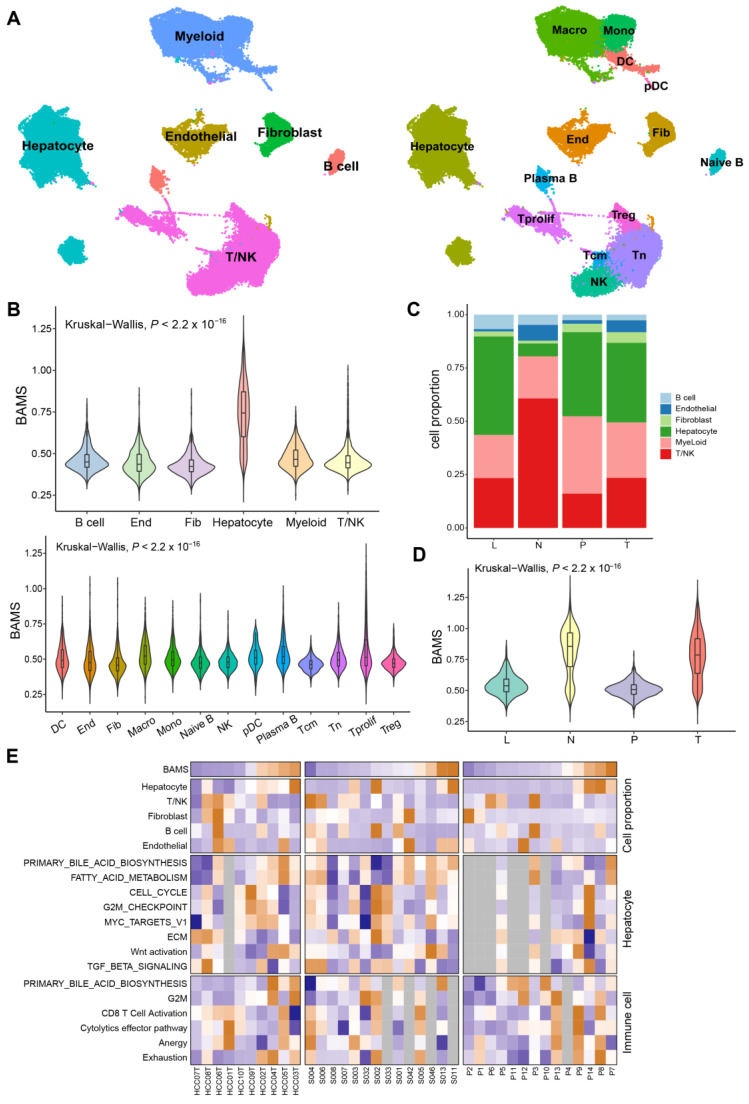
Single-cell RNA sequencing (scRNA-seq) analysis of HCC patients regarding BAMS in GSE149614. (**A**) The uniform manifold approximation and projection (UMAP) plot of major cell types (**left**) and subclusters (**right**). Macro: Macrophage; Mono: Monocyte; DC: Dendritic cell; pDC: Plasma dendritic cell; End: Endothelial cell; Fib: Fibroblast; T prolif: Proliferation T cell; Tcm: Central memory T cell; and Tn: Naïve T cell. (**B**) Violin plot of BAMS in major cell types (**top**) and subclusters except for hepatocytes (**bottom**). The statistical difference was compared via the Kruskal–Wallis test. (**C**) Stacked bar plots showing the percentages of major cell types in each tissue type. L: MLN metastatic lymph node; N: non-tumor liver; P: portal vein tumor thrombus; T: primary tumor. (**D**) Violin plot described the BAMS of hepatocytes in each tissue. (**E**) Clustered heatmap of related signatures across three datasets (GSE149614, GSE151530, and GSE156625). The samples are ordered by BAMS from the lowest to the highest from the color blue to orange, respectively. Grey rectangles indicate that the number of cells in the sample is less than 50.

Given the disparities in BA metabolism between tumor and non-tumor tissues, we sought to investigate whether similar differences persisted at the single-cell transcriptome level. After quality filtering using the R package “Seurat (v.4.2.0)”, approximately 70,000 single cells from GSE149614 were included and classified into six major clusters and 14 subclusters for further analysis (Figure 5A). Each cell sample was assigned a BAMS according to the ssGSEA algorithm. The highest BAMS was seen in hepatocytes and varied little in other cell types (Figure 5B), suggesting hepatocytes were the primary cells affecting BA metabolism. For stromal cells, macrophages and Plasma B had the highest BAMS compared to other cells, except for hepatocytes (Figure 5B). Non-tumor tissue (N) exhibited the significant depletion of hepatocytes and enrichment in T/NK cells compared to primary tumor tissue (T), metastatic lymph node (L), and portal vein tumor thrombus (P) in the composition of major cell types (Figure 5C). To be noted, in hepatocytes, BAMS was higher in the non-tumor tissue than in the tumor tissue, consistent with the findings in the tissue transcriptome data (Figure 1B), followed by L and P cells (Figure 5D). However, BAMS was most enriched in the tumor tissue when all cell types were considered (Supplementary Figure S4A). We then wondered whether this was related to the proportion of each cell type in the tumor tissue. Subsequently, 10 tumor tissue samples of GSE149614 were divided into high and low groups by the median cut-off of BAMS to investigate the differences in BA metabolism between cell types (Supplementary Figure S4B). As expected, hepatocytes were enriched in the high group, while the differences in other cell types were not significant (Supplementary Figure S4C). Furthermore, we assessed the associations between BAMS at the individual level of cell proportions and the enrichment of relevant pathways in hepatocytes and immune cells at the sample level (Figure 5E). The proportion of hepatocytes exhibited a trend similar to BAMS, whereas T/NK cells showed a roughly opposite pattern, and T cell exhaustion appeared to be positively correlated with BAMS. Moreover, the negative correlation between BAMS and CCS was found only in hepatocytes (Spearman R = −0.3, *p* < 0.01) (Supplementary Figure S4D).

### 2.6. Association between BA Metabolism-Related Genes and Cell Cycle-Related Genes

Since there was a negative connection between BA metabolism and cell cycle-related pathways, we defined a cell cycle gene set obtained from five gene sets and then screened using DEGs in HCC (Supplementary Table S2) to calculate the cell cycle-related gene enrichment score (CCS) of 368 HCC samples. BAMS was significantly negatively correlated with CCS (Spearman R = −0.52, *p* < 0.001, Supplementary Figure S5A), which was also found in the ICGC-LIRI cohort (Spearman R = −0.55, *p* < 0.001, Supplementary Figure S5B). To identify specific negatively related BA metabolism-related genes and cell cycle-related genes, we conducted a correlation analysis. The heatmap illustrated the top 30 negatively correlated BA metabolism-related genes and cell cycle-related genes (Supplementary Figure S5C). The prediction of transcription factors based on ChEA3 [27] (https://maayanlab.cloud/chea3/ (accessed on 20 October 2022)) was performed to explore potential TFs upstream co-regulating these genes (Supplementary Table S5). *MYBL2*, *TCF3*, *ETV4*, and *MYB* were found to be negatively correlated with BA metabolism-related genes and positively correlated with cell cycle-related genes. Conversely, *HNF4A*, *NR1I3*, *KLF15*, and *NR1I2* were positively correlated with BA metabolism-related genes while negatively correlated with cell cycle-related genes (Supplementary Figure S5D).

### 2.7. UDCA Inhibits Cell Cycle-Related Genes and Markedly Affects Cancer Cell Phenotype

**Figure 6:**
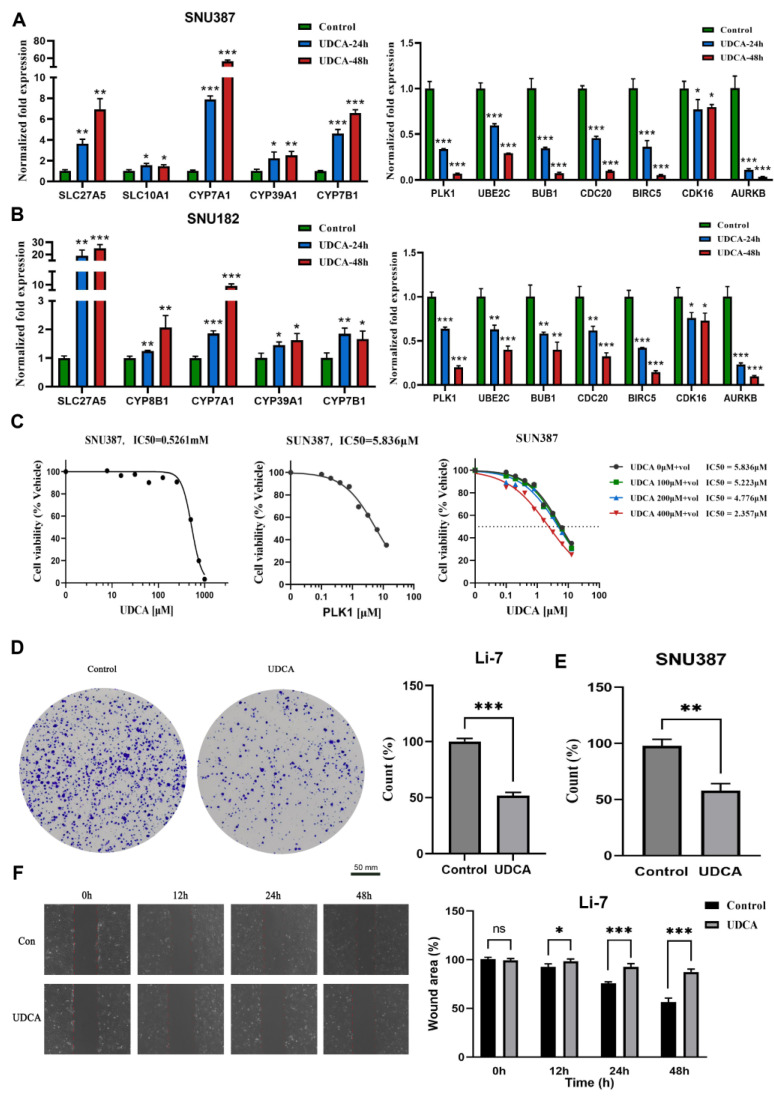
UDCA suppresses cell viability and migration in HCC cells. Relative cell cycle-related genes and BA metabolism-related gene levels using UDCA were analyzed via qPCR in SNU387 (**A**) and SNU182 (**B**). (**C**) Cell growth curve indicates the IC_50_ of UDCA (**left**), IC_50_ of volasertib (**middle**), and increasing concentrations of UDCA in a combination from 0 to 400 μM (**right**) in SNU387; “+vol” means add Volasertib, dashed line is IC_50_. Images of colony formation assays in Li-7 (**D**) and SNU387 (**E**). (**F**) Images of the wound-healing assay in 0, 12, 24, and 48 h (**left**), and counts of migrated tumor cells shown on the (**right**), Li-7, the scale bar was same as (**D**). Results are means ± SD of three independent experiments. (ns, not significant, * *p* < 0.05, ** *p* < 0.01, *** *p* < 0.001).

BAs are synthesized in the liver and degraded by the intestinal microbiota where they are further metabolized to secondary BAs [28]. UDCA is a secondary BA and has been implicated as an FXR inhibitor [29], and inhibition of the FXR in the ileum and liver promotes the synthesis of BAs. Since the negative correlation between BAMS and CCS was identified at the tissue transcriptome level, it was important to further inquire whether UDCA could also regulate the cell cycle in an in vitro cell experiment. As was depicted in Figure 6A, UDCA positively regulated the activity of BA metabolism-related genes, such as *SLC27A5*, *CYP7A1*, and *CYP7B1* (involved in BA synthesis), *SLC10A1* (transporter of conjugated bile salts from plasma into the hepatocyte), and *CYP39A1* (involved in the conversion of cholesterol to BAs). Nevertheless, UDCA inhibited the expression of cell cycle-related genes and the repression effect was enhanced with a time increase (*PLK1*, *UBE2C*, *BUB1*, *CDC20*, *BIRC5*, *p* <0.001, Figure 6A) in hepatoma cell lines SNU387. The above regulatory relationships were also found in Li-7 and HUH-7 cell lines (Figure 6B, Supplementary Figure S6A,B). These results further verified the negative relationship between BA metabolism and the cell cycle.

The IC_50_ of UDCA was determined in SNU387 cells as 0.5261 mM after 24 h of exposure across various concentrations (Figure 6C, left). Polo-like kinase 1 (PLK1) is a serine/threonine protein kinase involved in cell cycle regulation, and aberrant PLK1 signaling has been implicated in various cancers, including HCC [30]. To assess the antitumor activity, we combined UDCA with one PLK1 inhibitor (Volasertib). The IC_50_ of the PLK1 inhibitor was identified as 5.836 µM in SNU387 cells after 24 h of exposure across a concentration range (Figure 6C, middle). The results from the CCK8 assay indicated that the combination therapy (UDCA and Volasertib) exhibited a superior effect compared to monotherapy (Figure 6C, right). Moreover, we also found that UDCA repressed colony formation in several HCC cell lines (Figure 6D,E, Supplementary Figure S6C,D). The wound-healing experiment illustrated that UDCA slowed cell migratory ability compared to the control (Figure 6F, Supplementary Figure S6E–G).

In summary, UDCA demonstrated a promotion of the expression of genes associated with BA synthesis and exhibited a suppressive effect on cell cycle-related genes. Additionally, UDCA was observed to reduce cell viability, inhibit tumor migration, and decrease cell cloning in vitro. Importantly, it holds the potential to enhance cytotoxicity when combined with cell cycle inhibitors for potential use against HCC.

## 3. Discussion

**Figure 7:**
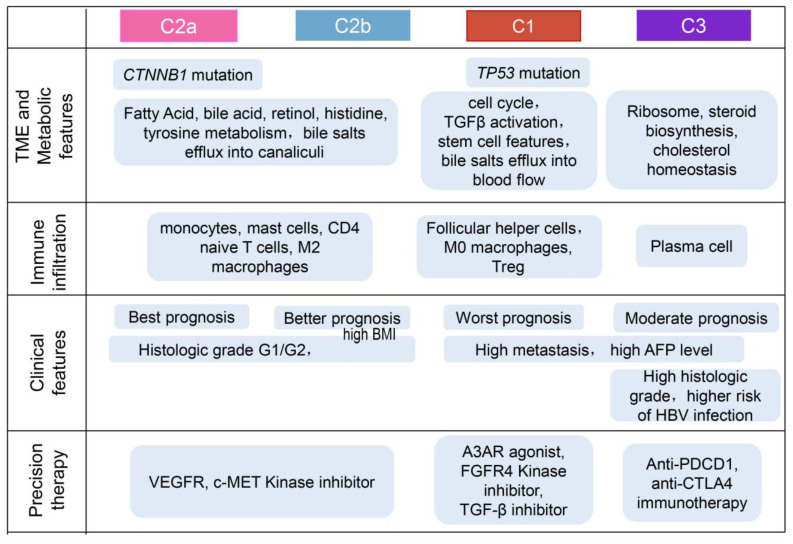
Overview of characteristics of comprehensive characterization of HCC subtypes.

Existing knowledge about perturbations in BA metabolism in HCC has primarily been derived from metabolomics studies based on serum samples, aiming to identify potential serum biomarkers related to BAs. However, a comprehensive exploration of the role of BA metabolism in HCC tissue samples, utilizing transcriptomic datasets, remains to be obtained. In this study, we collected a gene set related to BA metabolism to reconstruct the entire tumor’s composition and conducted integrated analyses to gain insights into the TME under the regulatory influence of BA metabolism. The identified four subtypes exhibited distinct molecular and histopathologic characteristics, therapeutic responses, and prognosis (Figure 7). Importantly, our analysis revealed a negative correlation between BA metabolism and cell cycle-related pathways, as demonstrated by the characteristics of the subtypes identified through unsupervised clustering based on BA metabolism-related genes.

The dysregulation of bile acid metabolism has been detected to be closely related to liver diseases and carcinogenesis [31]. We developed BAMS based on bile acid metabolism-related genes using the ssGSEA algorithm and proved that the BAMS was correlated with a clinical prognostic factor of HCC. Gene expression analysis has enabled the establishment of several HCC transcriptome classifications, and HCC can be generally categorized into two major subtypes: proliferative HCC and non-proliferative HCC [32,33,34]. Desert’s study identified two distinct phenotypes of non-proliferative HCC that were mutually exclusive, with favorable outcomes and preserved metabolic liver zonation programs [25]. Metabolic differences between proliferative and non-proliferative HCC have allowed the exploration of HCC subtypes with distinct characteristics from the metabolic point of view.

In this investigation, we delineated three distinct subtypes (C1, C2, ad C3) based on BA metabolism-related genes, delving into the correlations between metabolic characteristics, prognostic implications, transcriptomic features, immune infiltration, and clinical characteristics across these subtypes. Our findings unveiled that C1 exhibited a conspicuously active cell cycle reminiscent of proliferative HCC, featuring TGFβ activation, poorer prognosis, and a dearth of metabolic characteristics. On the contrary, C2 demonstrated associations with multiple metabolism pathways. Intriguingly, patients classified as the C2 subtype displayed higher BMI and improved OS (Figure 2). In a separate study on HCC subtyping, the results showed three subtypes and iClust1 had significantly worse prognosis than iClust2 and iClust3, but had lower overweight and fat patients compared with iClust2 and iClust3 [35]. Other previous research has indicated that obesity and high BMI contribute to increased liver cancer mortality and the incidence of primary liver cancer [36,37]. However, few studies have incorporated BMI indices into their investigations on liver cancer subtyping and BA metabolism, resulting in limited references and comparisons for the observations in our study. Therefore, our observations underscore the need to integrate clinical BMI data in future studies to comprehensively explore the association between obesity and the prognosis of liver cancer patients with dysfunctional BA metabolism. C2 could be further divided into C2a and C2b, resembling the PV and PP subtypes, respectively [25], being well differentiated and having the best prognosis of all subtypes. C2a carried more CTNNB1 mutations matching the PV type and *CTNNB1* subclass [23]. In another study, S3 HCC patients had a better prognosis than S1 and S2 HCC patients, and were well differentiated, and 50% of them carried *CTNNB1* mutations [21]. In our investigation, C2 displayed the highest resemblance to the S3 subtype, whereas C3 exhibited *AFP* overexpression and a closer association with the S1 subtype. On the other hand, C1 was linked to the S2 subtype, characterized by elevated serum AFP levels. Additionally, C3 appeared to manifest as an intermediate pattern between C1 and C2, featuring moderate metabolic and proliferative characteristics. Our single-cell analysis unveiled a primary association between BA metabolism levels and the proportion of hepatocytes, further emphasizing that the negative correlation between BAMS and CCS was discernible exclusively in hepatocytes.

Our study revealed identified transcription factors potentially regulating cell cycle and bile acid metabolism, respectively. MYBL2 has been implicated in promoting tumor progression by regulating the cell cycle in various cancers, including gastric cancer [38], prostate cancer [39], and also liver cancer [40]. MYB, known for its overexpression in cancer tissues, modulates stem cell properties and cell growth in cancers through the regulation of MiR-143 [41]. TCF3, with significantly elevated expression in tumor tissues, promotes cancer cell proliferation by regulating the cell cycle in liver cancer [42]. However, the existing literature on their negative correlation with bile acid metabolism is limited, necessitating further investigations to elucidate this mechanism. NR1I3 has been revealed to play crucial roles in coordinating bile-acid-activated gene expression programs, harmonizing regulatory activities in both the intestine and liver to control drug and bile acid transport [43]. Xenobiotic-sensing nuclear receptors NR1I2 and constitutive androstane receptor NR1I3 are known to play crucial roles in the classical synthesis and metabolism pathway of bile acids [44]. Over the past decade, studies have identified KLF15 as a crucial regulator of metabolism [45], showing that KLF15 enhances bile acid production in the liver, leading to increased nutrient uptake [46]. This suggests that KLF15 may emerge as a major therapeutic target for bile-acid-related metabolic diseases [47]. A study implicates HNF4A as the top upstream regulator of metabolism in liver cancer, particularly in a subtype of cholangiocarcinoma with high HNF4A and genes related to bile acid metabolism [48]. Despite these findings, the association between these genes and liver cancer progression through the regulation of bile acid metabolism remains poorly understood, necessitating further studies to fill this knowledge gap.

CDK4/6 inhibitors, such as palbociclib or ribociclib, in combination with letrozole have been approved as a first-line treatment for breast cancer [49,50]. However, the primary first-line agents for liver cancer predominantly remain tyrosine kinase inhibitors [51]. PLK1, which is less expressed during the mitotic phase of normal cells but highly expressed in most human cancer tissues, has been associated with poor prognosis in various cancers, including HCC [52,53]. PLK1 plays a pivotal role in chromosome segregation during the mitogen–anaphase transition and is crucial for cytokinesis. Additionally, PLK1 is indispensable for re-entering mitosis following recovery from G2 phase arrest induced by DNA damage, justifying its exploration as a potential therapeutic target for cancer [54]. Our previous studies have demonstrated that inhibiting PLK1 significantly impedes the cell cycle and proliferation of liver cancer cells [55]. Volasertib, among several PLK1 inhibitors, stands out as a highly selective inhibitor of the PLK family with the most significant effect on PLK1 [56]. UDCA, approved by the Food and Drug Administration (FDA) for treating primary biliary cirrhosis (PBC) since 1998, has garnered increasing attention for its potential anti-cancer properties over the last two decades [57]. However, limited information is available on how UDCA affects the growth and migration of HCC cells [58]. The prolonged and inappropriate use of UDCA may lead to liver damage and unwanted effects, such as pruritus, gastrointestinal discomfort, and fatigue [59,60]. Notably, a study has demonstrated a synergistic effect on the antitumor activity of HCC cells when UDCA is combined with sorafenib [61]. It appears that UDCA may be more promising in clinical use when combined with other drugs. Our study contributes additional perspectives on the molecular mechanisms of HCC by identifying a negative regulatory relationship between BA metabolism and the cell cycle through tissue and single-cell transcriptome data as well as cell line experiments. Furthermore, we validated the inhibitory effects of UDCA on the proliferation and migration of HCC cells. The in vitro results indicated that the combination of UDCA and volasertib exhibited a synergistic inhibition of cell viability. In this study, we revealed the potential tumor-inhibitory properties of UDCA and suggested a plausible potential tumor inhibition strategy involving the combination of UDCA with PLK1 inhibitors. This necessitates further research, which needs to be prioritized to validate the potential clinical efficacy of UDCA.

## 4. Materials and Methods

### 4.1. Sample Collection and Data Processing

Clinical and survival information of the training cohort TCGA-LIHC with 374 HCC and 50 adjacent non-tumor samples was accessed from TCGA. In total, 368 HCC samples were further used for analysis after excluding those with no clinical information. The gene expression matrix and gene somatic mutation data were downloaded from the UCSC XENA (https://xenabrowser.net/ (accessed on 20 May 2022)). The validation cohorts (GSE14520, GSE36376, and GSE25097) and the single-cell sequencing dataset (GSE149614, GSE151530, and GSE156625) were retrieved from the Gene Expression Omnibus (GEO) dataset (http://www.ncbi.nlm.nih.gov/geo (accessed on 25 May 2022)). Another validation cohort LIRI was obtained from the International Cancer Genome and Consortium (ICGC) database (https://dcc.icgc.org/ (accessed on 13 June 2022)). The RNA-seq raw read count from the TCGA database was converted to transcripts per kilobase million (TPM) and further log2 transformed. Gene sets used for gene set variation analysis (GSVA) were obtained from the Molecular Signature Database v. 7.5 (MSigDB). Differential expression genes analysis was conducted using R package “DESeq2 (v. 1.42.0)” and absolute log2 fold change (FC) > log2 (1.5) and adjusted *p* < 0.01 were considered as differentially expressed genes (DEGs). Detailed information on datasets above is described in Supplementary Table S6.

### 4.2. Gene Set Variation Analysis

GSVA implemented with the R package “GSVA (v.1.38.2)” was used to calculate the GSVA enrichment scores of pathways with at least 10 overlapping genes for each sample. The gene sets were obtained from the MSigDB database and 50 cancer hallmark gene sets. The TCGA-LIHC expression matrix was subjected to the GSVA algorithm to calculate the ssGSEA scores for BA metabolism-related BAMS and cell cycle-related gene enrichment score (CCS). Differential pathway analysis was implemented using the R package “limma (v. 3.58.1)”.

### 4.3. Identification of HCC Subtypes

The TCGA-LIHC cohort with clinical information, including 368 HCC samples, was used to identify the subtypes using the NMF consensus cluster method implemented in the R package “NMF (v. 0.21.0)” [62]. Ninety-five BA metabolism-related genes screened by DEGs between HCC and non-tumor samples were subjected to NMF for unsupervised consensus clustering. The rank survey profiles of the cophenetic score and silhouette width indicated a three-subtype solution for HCC samples. A T-distributed stochastic neighbor embedding (t-SNE)-based approach was used to validate the subtype clustering using the mRNA expression data of 95 BA metabolism-related genes. The C2 cluster was further identified as the C2a and C2b subtypes using the same algorithm.

### 4.4. Characterization of HCC Subtypes by Previous Molecular Signatures

To compare our four subtypes with previously identified molecular subtypes of HCC, 368 HCC samples were classified according to molecular signatures from previous studies. Seven published HCC classifications were collected for comparison: cholangiocarcinoma-like HCC (CLHCC) [19], Chiang classification [23], metastasis gene signature [22], activation and silencing of the Hippo pathway [18], Hoshida subtypes [21], Boyault classification [63], and 65-gene-based risk score (65RS), calculated via the method described in this study [24]. Signatures for calculating ssGSEA score to explore the correlation of distinctive characterization between subtypes were derived from previous studies (Supplementary Table S7).

### 4.5. Single-Cell RNA Sequencing (scRNA-Seq) Analysis

The scRNA-seq dataset GSE149614 contained about 70,000 single-cell transcriptomes for 10 HCC patients from four tissues: primary tumor (T), portal vein tumor thrombus (P), metastatic lymph node (L), and non-tumor liver (N), which was processed using R package “Seurat (v.4.2.0)” for normalization. Cells expressing less than 300 molecular identifiers and more than 20% of mitochondrial reads were screened out. The top 2000 variable genes were used for further clustering and the first 50 principal components were further dimensionalized using UMAP. Cell-type annotation referred to CellMarker (http://xteam.xbio.top/CellMarker/ (accessed on 19 October 2022)) [64]. The BAMS of the scRNA-seq dataset was calculated using the ssGSEA algorithm.

### 4.6. Reagents

Ursodeoxycholic acid (UDCA) was purchased from MedChemExpress (Cat. No. HY-13771, Monmouth Junction, NJ, USA). Volasertib (PLK1 inhibitor) was purchased from TOPSCIENCE (Cat. No. T6019, Shanghai, China). UDCA and volasertib were dissolved in dimethyl sulfoxide (DMSO) with final concentrations of 50 mM and 10 mM, respectively.

### 4.7. Cell Culture

All tumor cells were obtained from American-type culture collection (ATCC), and human hepatoma cell lines SNU387, SNU182, Li-7, and HUH7 were cultured in a medium containing 10% fetal bovine serum (Gibco, Grand Island, NY, USA) at 37 °C in an incubator with 5% CO_2_.

### 4.8. Cell Counting Kit-8 (CCK-8) Assay

The tumor with 10,000 cells/per well was seeded in 96-well plates and cultured at 5% CO_2_, 37 °C, and then tumor cells were treated with UDCA and Volasertib for 24 h in the logarithmic growth phase. The CCK8 assay was performed using a CCK8 kit following the manufacturers protocol (GK10001, GLPBIO, Montclair, NJ, USA) to detected cell viability.

### 4.9. Migration Assays

Tumor cells (2 × 10^5^ cells/well) were seeded in 6-well plates and allowed to adhere overnight. A cell scratch spatula was used to make a scratch in the cell monolayer when the culture reached 90–95% confluence. Digital images were taken at the beginning of the experiment (0 h), which was considered 100%. Tumor cells were incubated with UDCA at ½ × IC50 concentrations, and the migration of tumor cell scratch was observed at 12 h, 24 h, 36 h, or 48 h using an inverted fluorescence microscope (MF53-N, MSHOT, Guangzhou, China). ImageJ (v.1.8.0) was used to analyze the scratched area in the scratch assay quantitatively.

### 4.10. Colony Formation Assay

Cell proliferation capacity and cellular clonogenic potential were assessed using the clone formation assay. Tumor cells were seeded at a density of 1000–5000 cells/well in 6-well plates and allowed to adhere overnight. Then, UDCA was added and incubated at 1/2 × IC50 concentrations for 24 h. Tumor cells were rinsed three times with PBS and further grown for 7–14 d. The samples were stained with 1% crystal violet for 10 min after being fixed with 4% methanol for 30 min and then visualized using an inverted fluorescence microscope (MF53-N, MSHOT, Guangzhou, China). Immediately after, the images were analyzed using ImageJ (v.1.8.0). 

### 4.11. qRT-PCR Analysis

The total RNA was extracted from samples using TRIzol and reverse-transcribed using the Evo M-MLV RT Kit with gDNA Clean for qPCRII (AG11711, Accurate Biotechnology, Changsha, China) according to the manufacturer’s protocol. qRT-PCR reactions were carried out using SYBR Green Premix Pro Taq HS qPCR Kit (AG11701, Accurate Biotechnology, Changsha, China) and QuantStudio 1 (Appliedbiosystems by Thermo Fisher Scientific, Shanghai, China). The relative expression was calculated via the 2^−ΔΔCt^ method [65], and qRT-PCR primer sequences are listed in Supplementary Table S8.

### 4.12. Statistical Analysis

Statistical analyses and visualization were performed using R language software, version 4.1.2. Statistical significance was analyzed using the Wilcoxon test for the comparison of two groups. For comparisons between more than three groups, one-way analysis of variance and the Kruskal–Wallis test were performed. Contingency table variables were analyzed using the chi-square test. Differences were considered statistically significant at *p* < 0.05. All correlations were performed using Spearman correlation analysis. Kaplan–Meier analysis was performed to estimate the overall survival (OS) rates and disease-free survival (DFS) rates, and the difference in survival curves between BAMS-high and -low groups was compared via the log-rank test through R package “survival (v. 2.38_2)”. Statistical analyses for HCC cell line experiments were performed using Student’s *t*-test in the GraphPad Prism 9.4.1 software, and all of the experiments were repeated at least three times.

## 5. Conclusions

In summary, our investigation unveiled a downregulation of bile acid metabolism-related pathways in HCC tissue samples compared to non-tumor tissue samples. Furthermore, a low level of bile acid metabolism was linked to poor prognosis and advanced disease progression in HCC patients. By comparing our identified subtypes with previously established classifications, we gained additional insights into the molecular features of HCC. We introduced a novel perspective suggesting that HCC can be broadly categorized into proliferative and non-proliferative subtypes based solely on bile acid metabolism-related genes. Through comprehensive analyses of tissue and single-cell transcriptome data, as well as cell line experiments, we identified a negative correlation between bile acid metabolism and cell proliferative features. These findings highlight the potential significance of UDCA or other effective bile acid-based agents in combination with inhibitors targeting the cell cycle as a promising new strategy for HCC treatment.

## Data Availability

The datasets analyzed in the study are available in the public data repository TCGA, GEO, and ICGC. The other data are available from the corresponding author on reasonable request. More information presented in the study is described in the Supplementary Material.

## Supplementary Materials

The following supporting information can be downloaded at https://www.mdpi.com/article/10.3390/ijms25020919/s1.
