# Supplementary material for: Establishment and Validation of Novel Prognostic Subtypes in Hepatocellular Carcinoma Based on Bile Acid Metabolism Gene Signatures Using Bulk and Single-Cell RNA-Seq Data
Source: Int J Mol Sci. 2024 Jan 11;25(2):919. doi: 10.3390/ijms25020919 (PMC10815120; doi:10.3390/ijms25020919)
Supplement: Supplementary file 1 [file ijms-25-00919-s001.zip › Caption of Supplementary materials.pdf]

Supplementary material caption:

Figure S1. GSVA analysis of bile metabolism (A) and differential pathways analysis between tumor and non-tumor samples in the validation cohorts (B). (GSE14520 (220 non-tumor, 225 tumor), GSE25097 (243 non-tumor, 268 tumor), GSE36376 (193 non-tumor, 240 tumor)). (C) Kaplan–Meier curves of OS for BAMS-high and BAMS-low in GSE14520.

Figure S2. (A) BAMS in patients with different clinicopathologic features, tumor stage T stage, and histological stage. (B) NMF clustering using 95 BAs metabolism-related genes. Cophenetic correlation coefficient for k = 2-6 is shown. (C) t-SNE analysis in HCC colored by the three subtypes. (D) Spearman correlation analysis between BAM and other altered pathways. Red indicates positive correlation with BAM, blue for negative correlated. (E) Heat map of altered pathways in the three subtypes of ICGC-LIRI cohort. (ns, not significant, **P < 0.01, ***P < 0.001, ****P < 0.0001).

Figure S3. The different gene expression between subtypes.

Figure S4. Spearman correlation analysis between BAMS and CCS in the TCGA-LIHC cohort (A) and the ICGC-LIRI cohort (B). (C) Heat map of correlation between BAs metabolism-related genes and CCGs. Red indicates positive correlation and blue indicates negative correlation, with the shape of the pie representing the correlation value. (D) Volcano plot demonstrating the correlation of BAs metabolism-related genes and CCGs co-regulated by the potential TFs. Circle representing BAs metabolism-related genes and triangle for CCGs, red indicating positive correlation with TF, with blue denoting negative correlation.

Figure S5. (A) BAMS of all cell types in each tissue. (B) Proportion of each cell subtypes in the BAMS high and low group. (C) Box plot of hepatocyte, T/NK, myeloid, monocyte, Tn and NK cell fraction in the BAMS high and low group. (D) Spearman correlation analysis between BAMS and CCS of major cell types.

Figure S6. Relative cell cycle-related genes and BAs metabolism-related genes levels using UDCA analyzed by qPCR in Li-7 (A) and HUH-7 (B). Images of colony formation assays shown the counts of focus in HUH-7 (C) and SNU182 (D). (E) (F) (G) Images of the wound-healing assays of the counts of migrated tumor cells compared to the con-trol in SNU182, SNU387, HUH-7.

Table S1: 236 gene sets used for GSVA.

Table S2: BAs metabolism-related genes and cell cycle related gene sets.

Table S3: Results of differential pathways analysis conducted by R package "limma".

Table S4: 90 genes for constructing classifier.

Table S5: List of potential transcription factors.

Table S6: Datasets detailed information.

Table S7: Signatures for calculating ssGSEA score to explore the correlation of distinc-tive characterization between subtypes.

Table S8: qRT-PCR primer sequences.
