# Supplementary figures and images for: Establishment and Validation of Novel Prognostic Subtypes in Hepatocellular Carcinoma Based on Bile Acid Metabolism Gene Signatures Using Bulk and Single-Cell RNA-Seq Data
Source: Int J Mol Sci. 2024 Jan 11;25(2):919. doi: 10.3390/ijms25020919 (PMC10815120; doi:10.3390/ijms25020919)

A

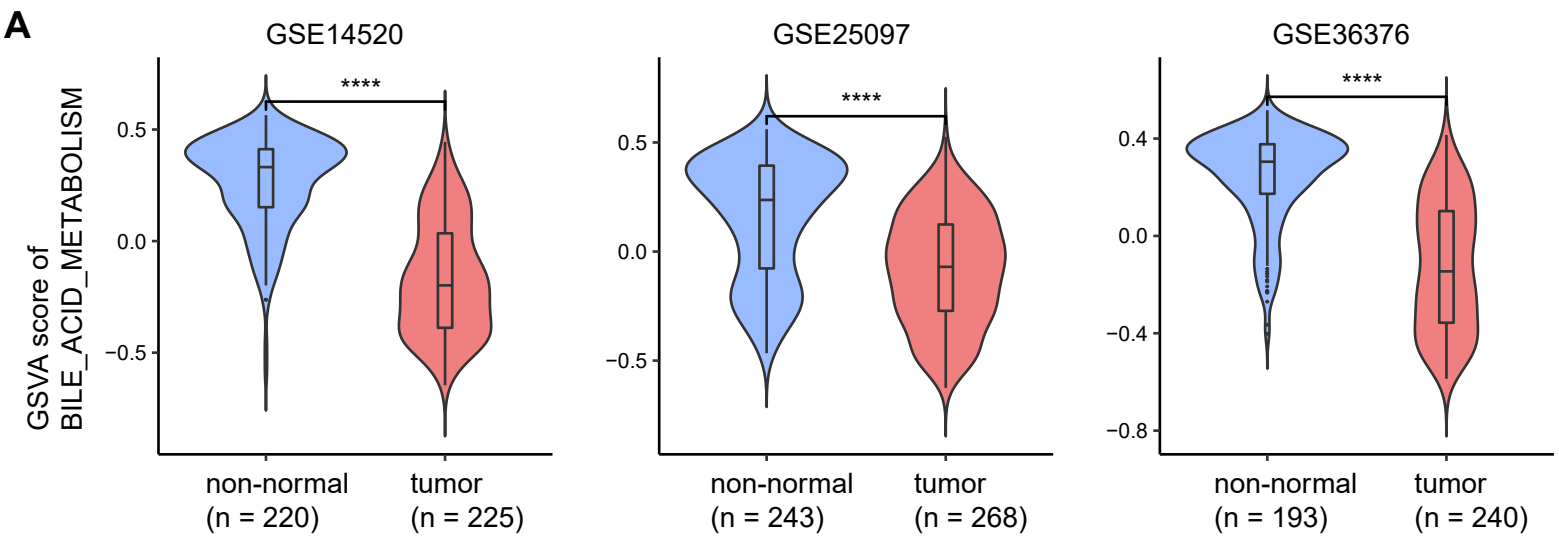

B

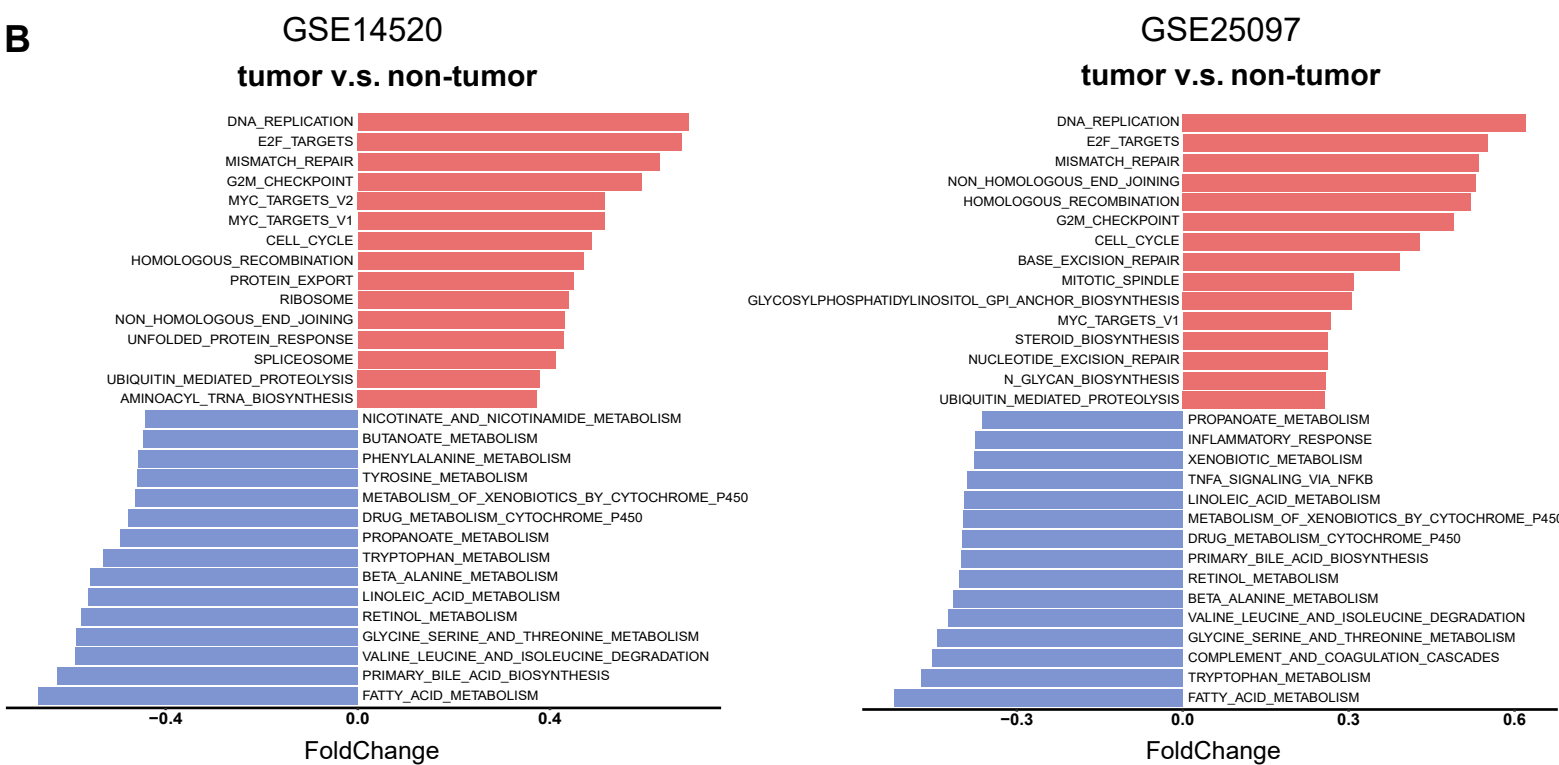

C

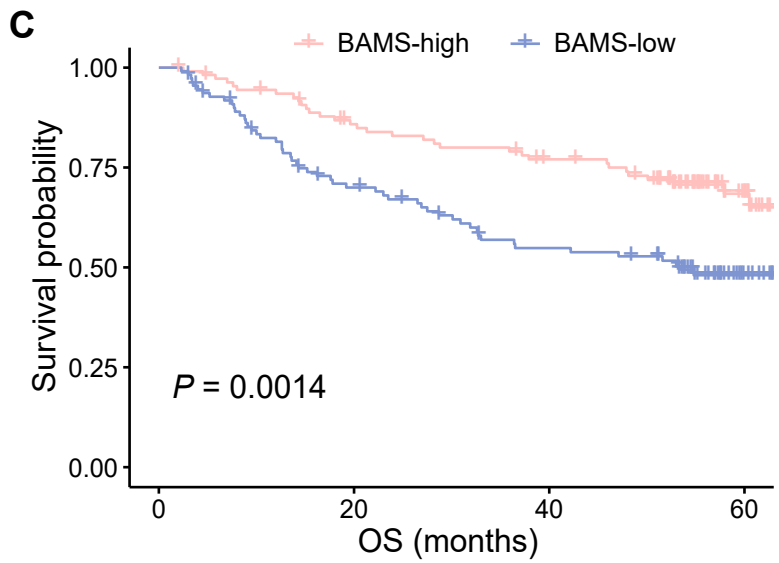

Supplement: Supplementary file 1 [file ijms-25-00919-s001.zip › FS1.pdf]

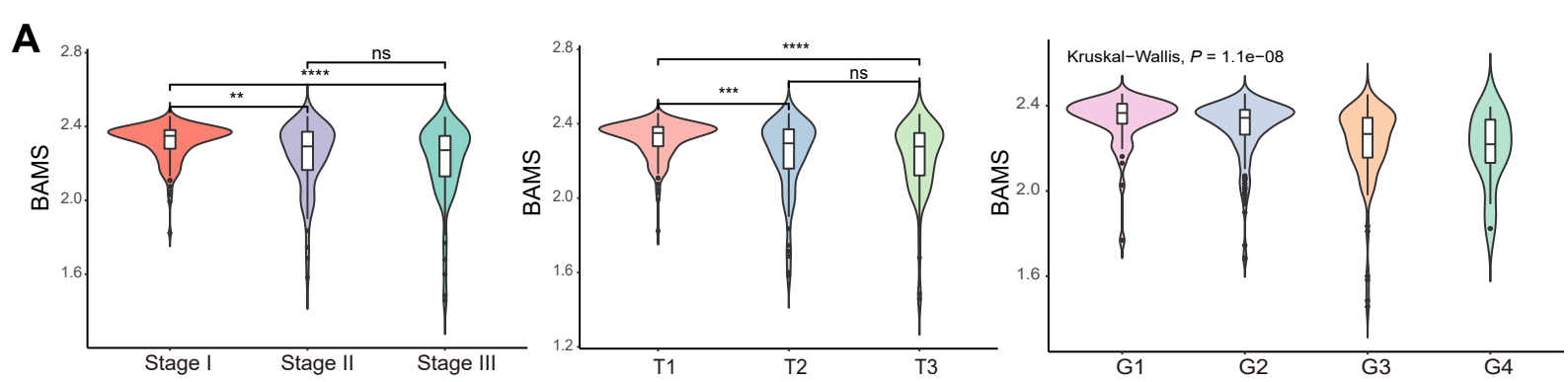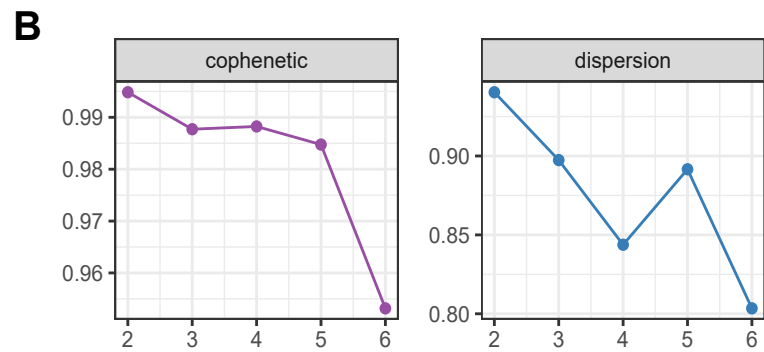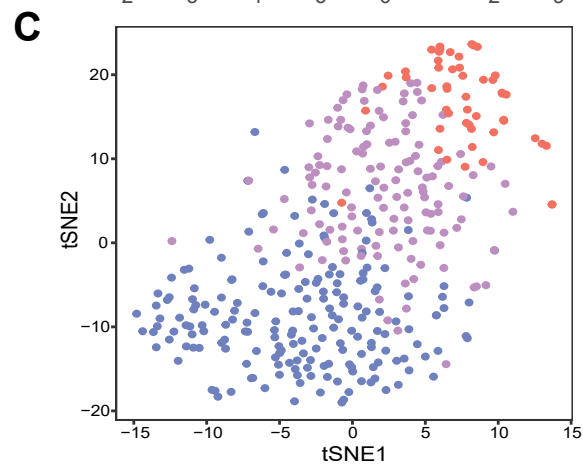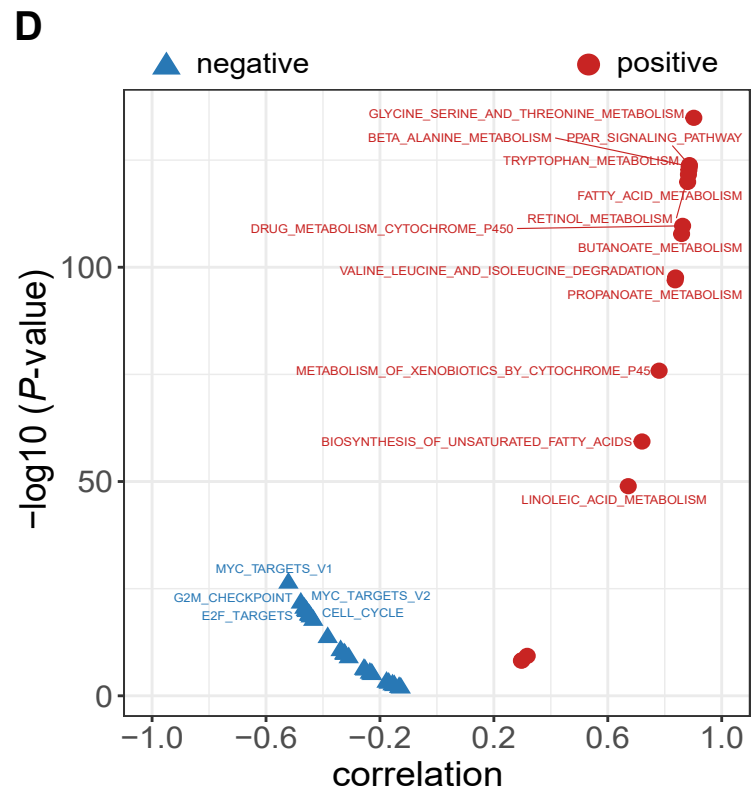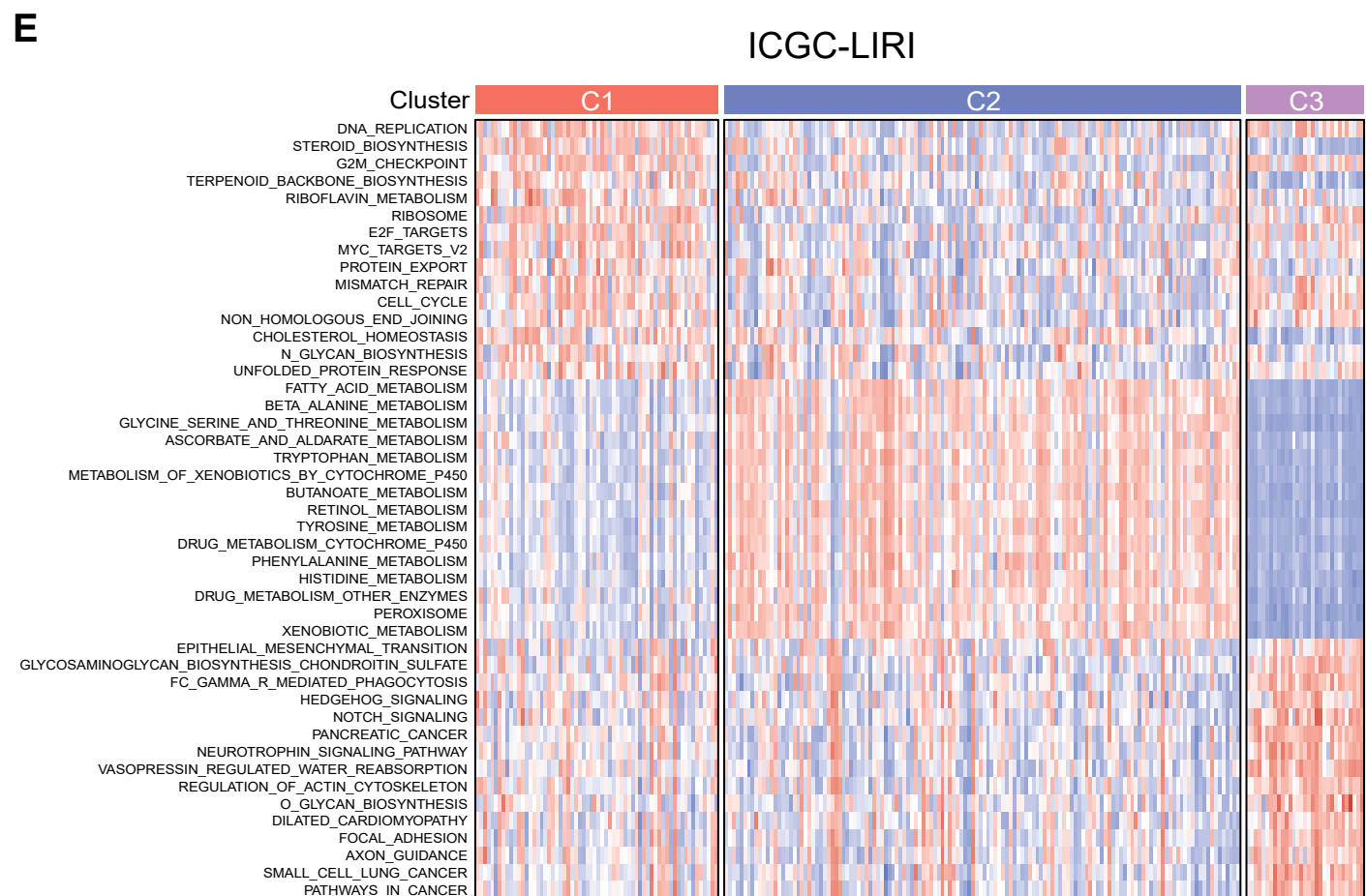

Supplement: Supplementary file 1 [file ijms-25-00919-s001.zip › FS2.pdf]

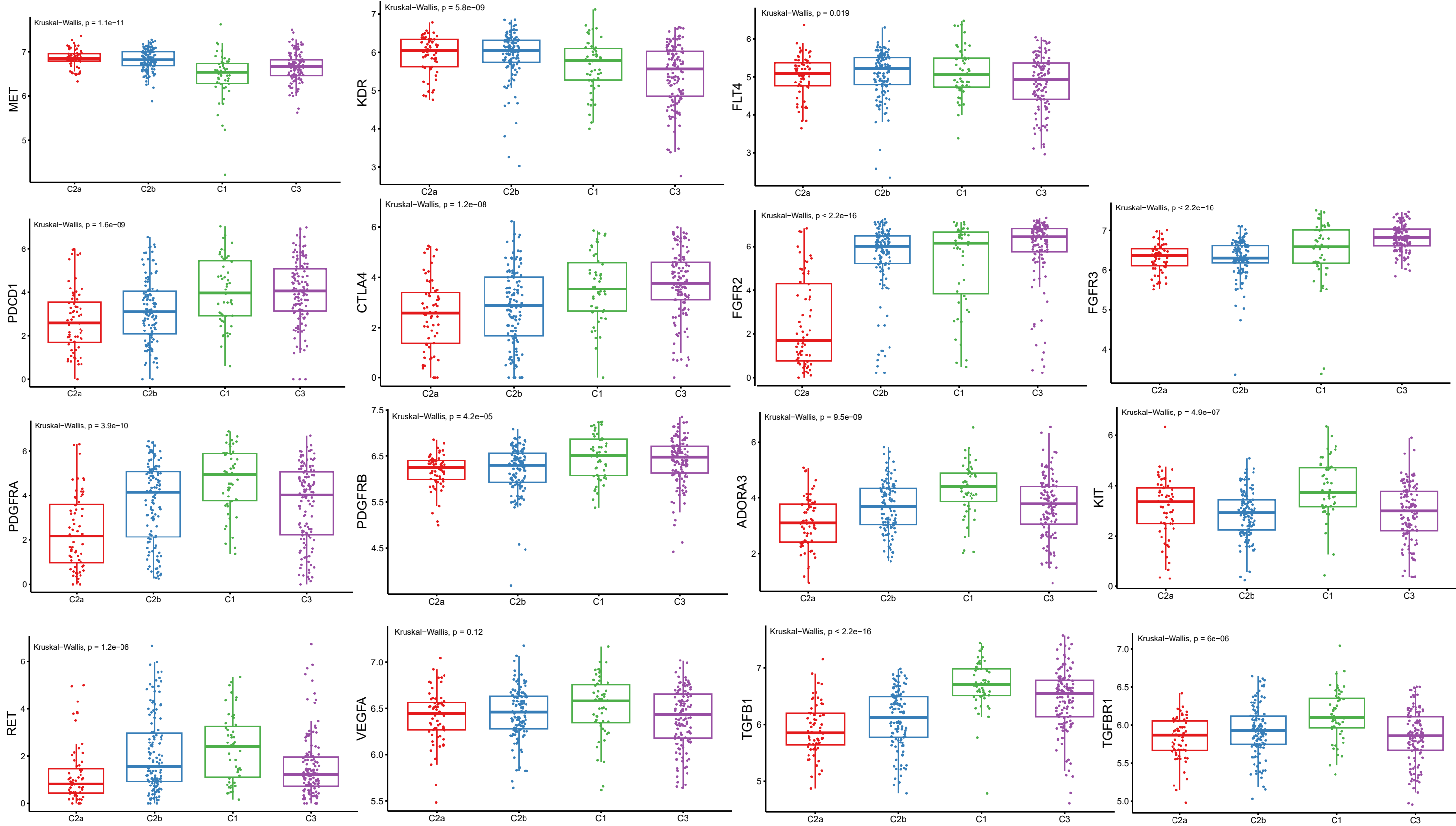

Supplement: Supplementary file 1 [file ijms-25-00919-s001.zip › FS3.pdf]

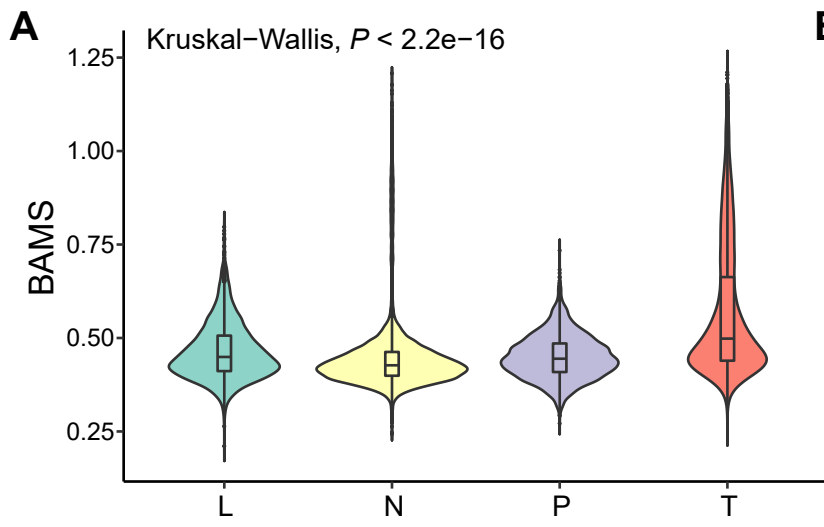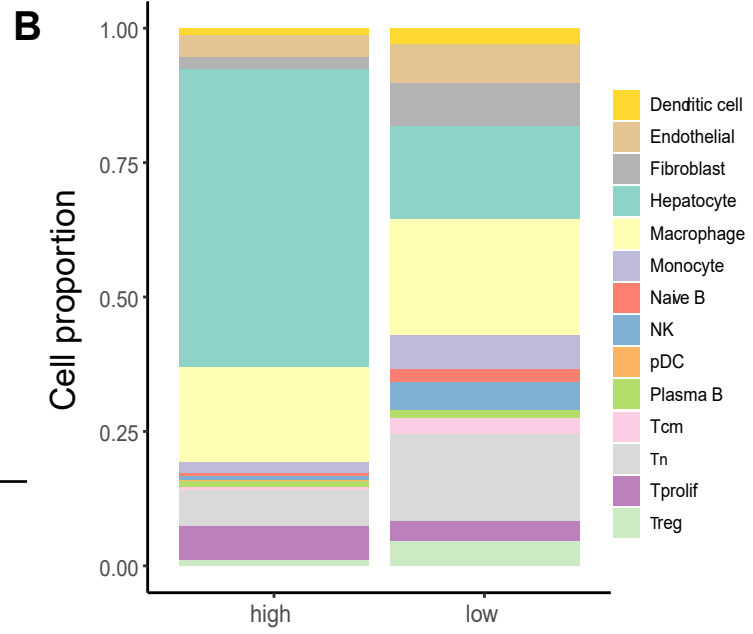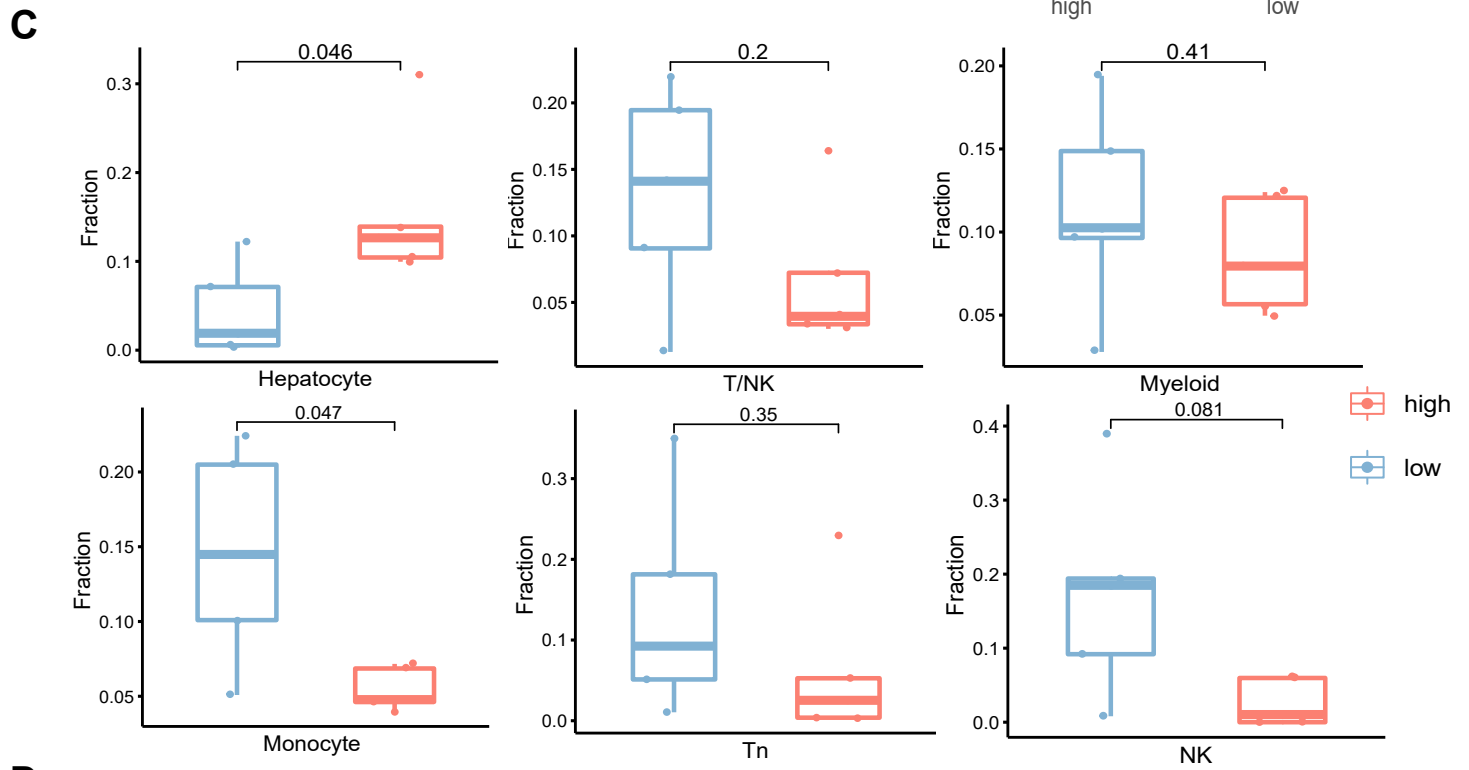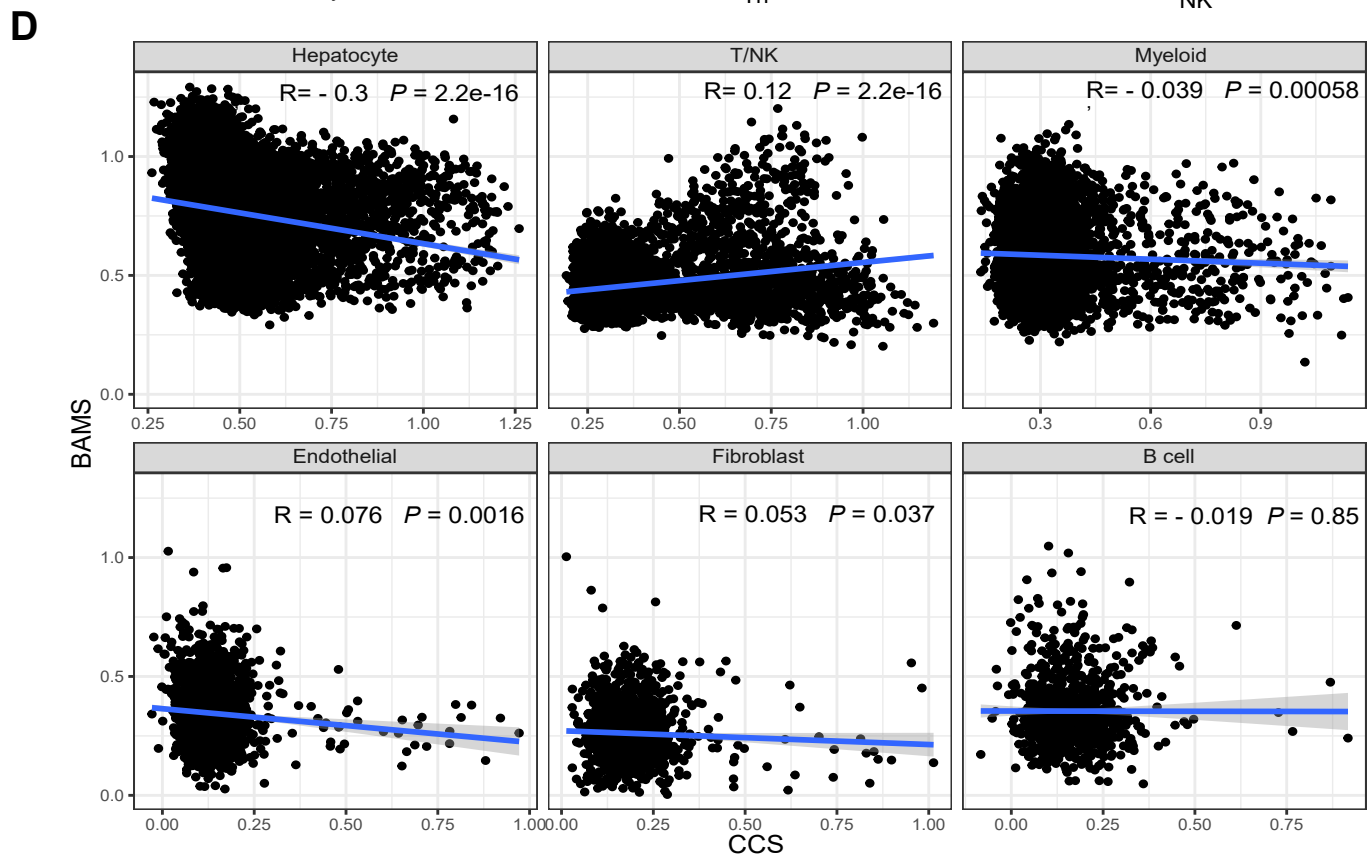

Supplement: Supplementary file 1 [file ijms-25-00919-s001.zip › FS4.pdf]

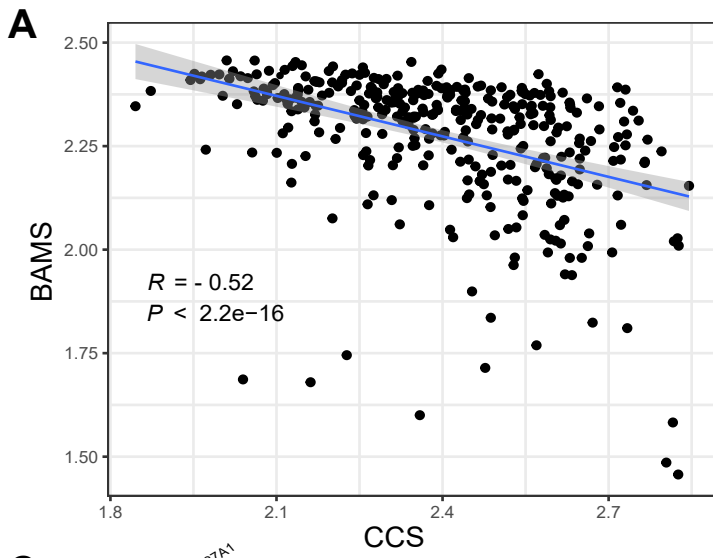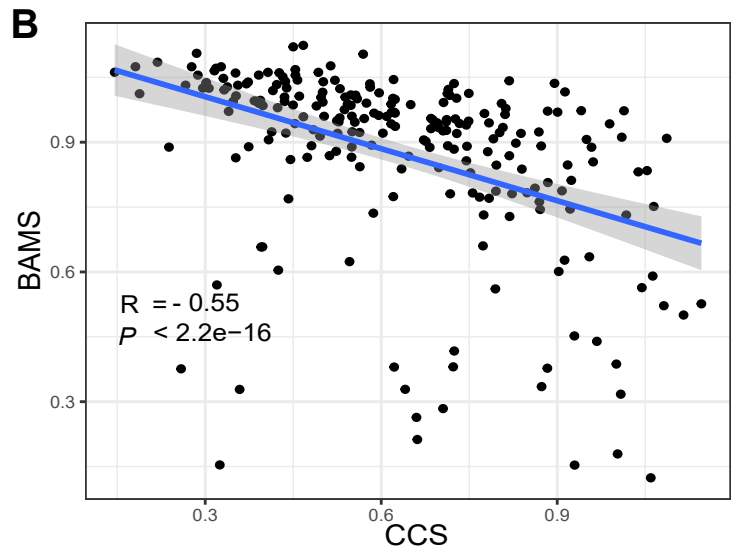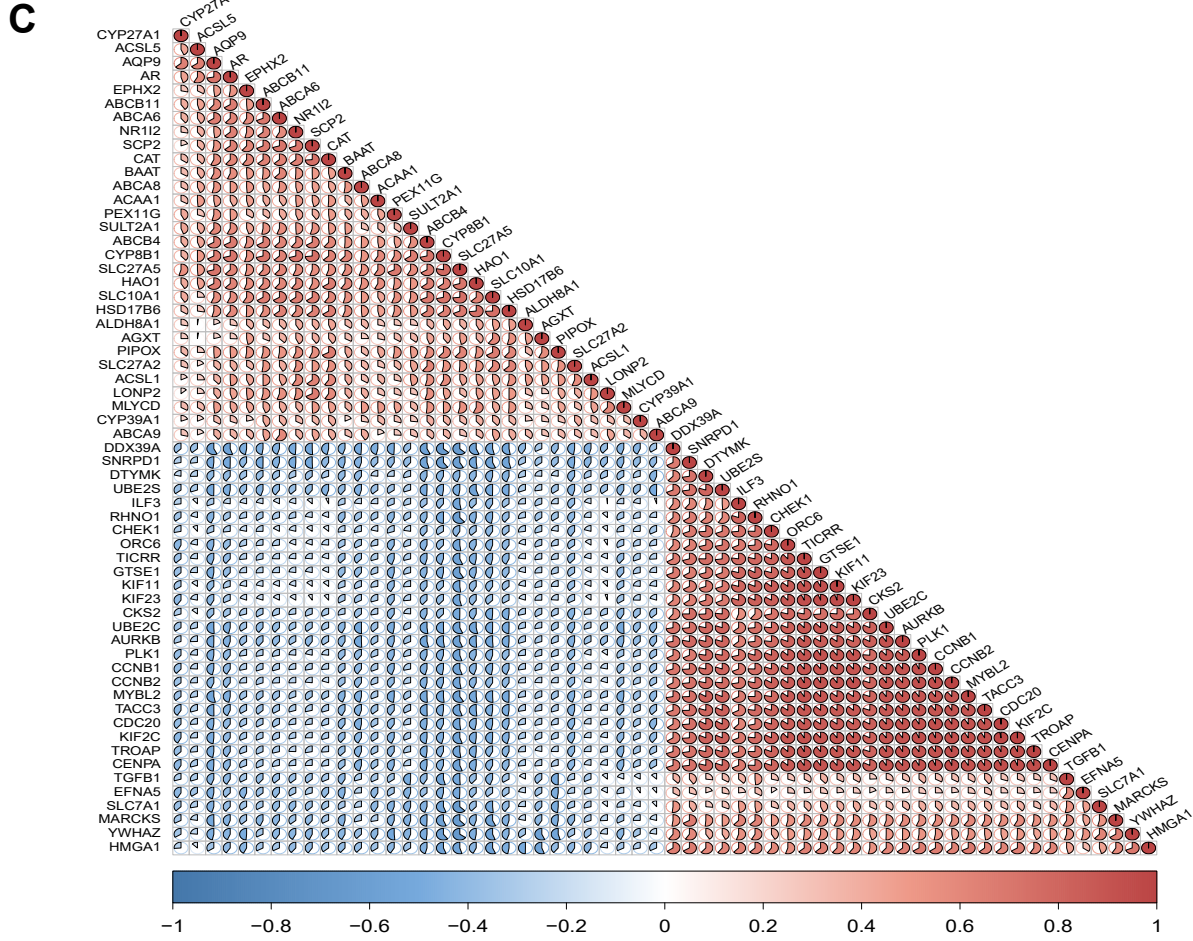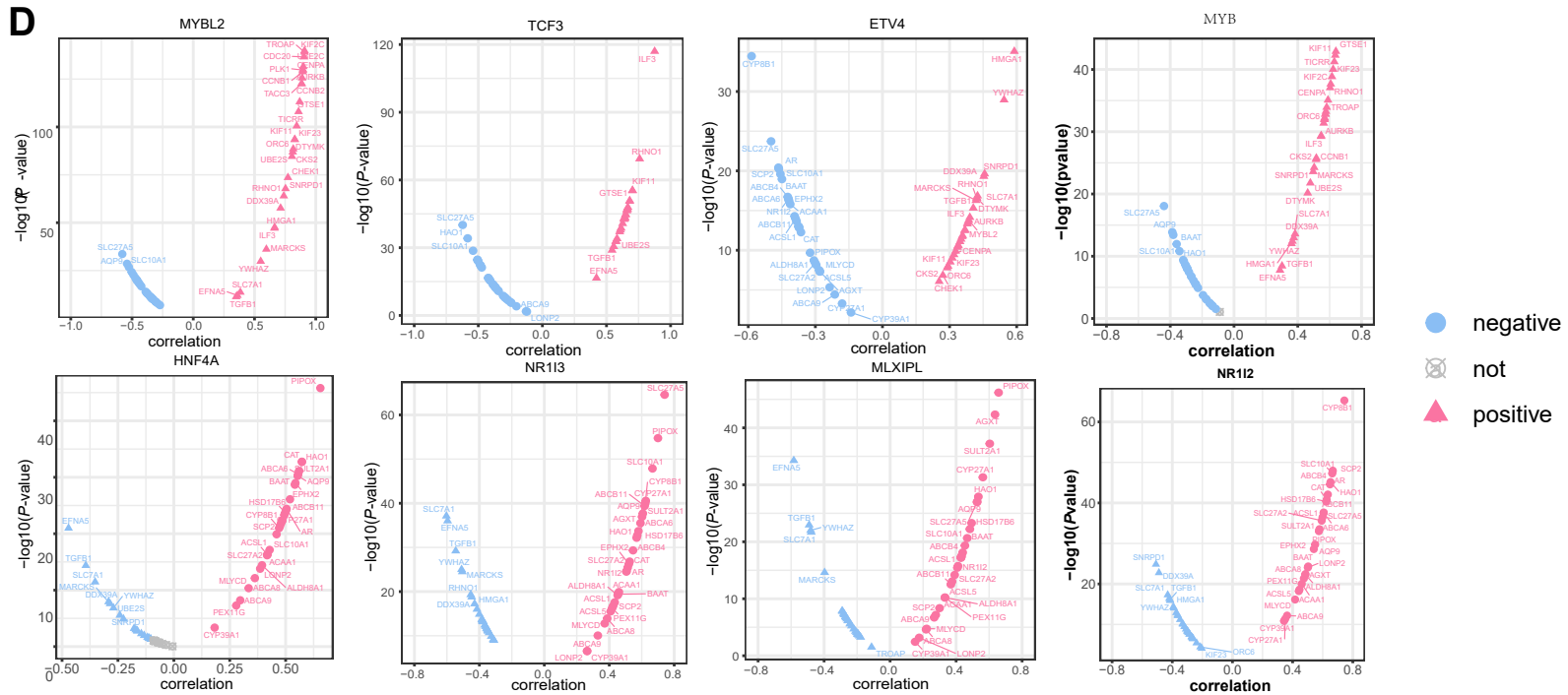

Supplement: Supplementary file 1 [file ijms-25-00919-s001.zip › FS5.pdf]

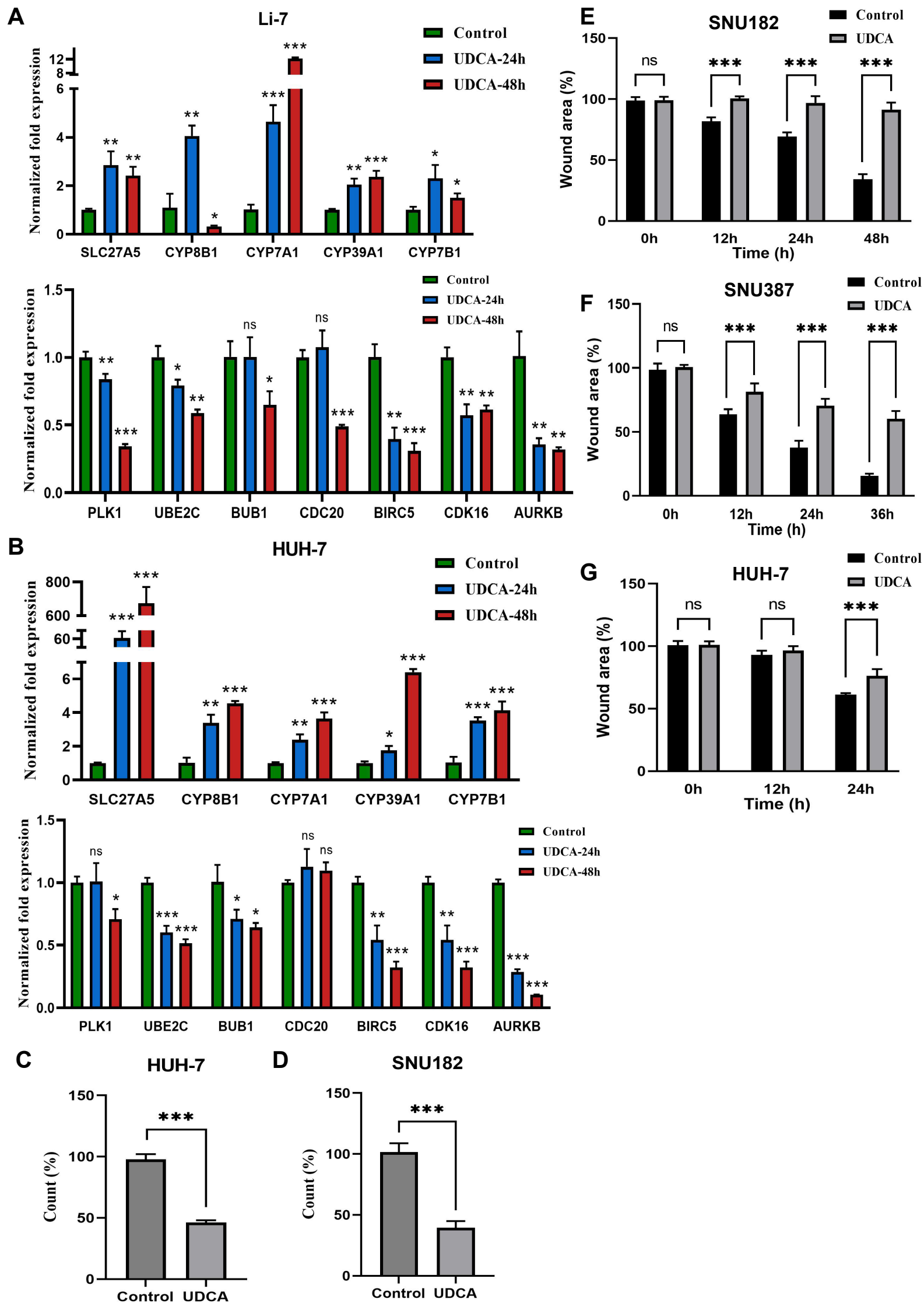

Supplement: Supplementary file 1 [file ijms-25-00919-s001.zip › FS6.pdf]
